# Psychological effects of hybrid SCMC with mobile device management: distraction, classroom atmosphere, and foreign language anxiety

**DOI:** 10.3389/fpsyg.2026.1775057

**Published:** 2026-03-25

**Authors:** Xuecheng Liu, Jiabei Zhu

**Affiliations:** 1 Institute of Global Humanities, Nanjing University, Nanjing, China; 2 School of General Education, Wanjiang University of Technology, Ma’anshan, China

**Keywords:** mobile device management (MDM), hybrid SCMC, foreign language anxiety, digital distraction, classroom atmosphere, mobile learning, technology-mediated learning, attention regulation

## Abstract

**Introduction:**

This study examines how mobile media regulation influences learners’ psychological experiences in hybrid synchronous computer-mediated communication (SCMC) environments, by introducing an integrated hybrid SCMC–Mobile Device Management (MDM) configuration as a unified instructional approach and empirically examining its psychological effects. Focusing on attention, distraction, and foreign language anxiety, it investigates whether Mobile Device Management (MDM) can shape learners’ affective and cognitive responses by regulating mobile device use during mediated interaction. Rather than evaluating instructional design, the study explores how technological conditions influence psychological processes underlying classroom engagement and interaction quality.

**Methods:**

A self-contrast study was conducted with 60 second-year undergraduate students (M age = 20.6, SD = 0.743) from a mid-tier university in Ma’anshan, China. The sample included 24 females and 36 males, all non-English majors enrolled in a compulsory English course. Participants experienced three instructional modes: traditional face-to-face instruction, hybrid SCMC without device regulation, and hybrid SCMC with MDM.

**Results:**

The results indicate that hybrid SCMC with MDM significantly improves classroom focus, reduces digital distraction, and enhances overall learning experience compared with the other conditions. However, the direct effect of MDM on foreign language anxiety is limited.

**Discussion:**

These findings suggest that MDM shapes learners’ psychological experiences primarily by regulating attention rather than directly modulating emotional responses. MDM helps regulate smartphone-related distraction, thereby creating more favorable attentional conditions for the effective implementation of hybrid SCMC. By clarifying how mobile media constraints shape learners’ psychological experiences, particularly through the integration of communicative affordances and attentional regulation, this study contributes to understanding media-mediated interaction in educational contexts and highlights how psychological mechanisms can inform the design of more effective hybrid learning environments.

## 1 Introduction

The rapid advancement of mobile technology has transformed contemporary educational settings, reshaping how students allocate attention, experience emotion, and engage in classroom interaction. With the widespread adoption of bring your own device (BYOD) practices, mobile devices have become integral to instructional environments, creating both opportunities for enhanced communication and challenges related to distraction and attentional control. In this context, understanding the psychological impacts of mobile technology use has become increasingly important. Mobile Device Management (MDM) systems offer a practical means of structuring mobile-mediated environments by regulating device use in ways that may influence learners’ attention, emotional states, and classroom experience.

In foreign language learning, learner psychology plays a decisive role in shaping participation and performance. Foreign language anxiety (FLA) has been consistently identified as a major factor that hinders learners’ willingness to communicate and engage in classroom activities. Previous studies have shown that hybrid synchronous computer-mediated communication (SCMC) can reduce FLA by creating a less threatening communicative environment and supporting student participation. From a psychological perspective, these benefits arise from changes in social presence, perceived evaluation, and self-expression afforded by text-based communication. However, the increasing reliance on mobile devices in SCMC contexts also introduces new psychological challenges, particularly internet-related distraction, which may weaken these positive effects.

Against this background, the present study investigates the psychological mechanisms through which MDM may influence learning experiences in hybrid SCMC environments. Rather than treating MDM as a pedagogical intervention alone, this research conceptualizes it as a contextual factor that shapes learners’ attentional regulation and emotional experience during mobile-mediated interaction. Specifically, the study examines whether the implementation of MDM can reduce foreign language anxiety and digital distraction while improving perceived classroom atmosphere and overall learning experience.

Using a self-contrast experimental design across multiple teaching modes, this study seeks to provide empirical evidence on how technological conditions affect learners’ psychological responses in instructional settings. By clarifying the psychological pathways through which mobile technologies influence learning, the findings aim to inform educational practice while contributing to a deeper understanding of media-related psychological processes in learning environments.

## 2 Literature review

### 2.1 Foreign language anxiety and synchronous computer-mediated communication

Foreign language anxiety (FLA) is a significant emotional barrier that can hinder language learning progress (MacIntyre, 2017; Toyama and Yamazaki, 2021). Horwitz et al. (1986) developed the Foreign Language Classroom Anxiety Scale (FLCAS) to assess FLA, identifying three primary sources: communication apprehension (anxiety and shyness when expressing with a foreign language), test anxiety (performance fears and concerns about exam failure), and fear of negative evaluation (worries about judgment and negative feedback). Since its introduction, FLCAS has become a widely used tool for assessing FLA in educational settings. Meta-analytic and empirical evidence has consistently shown a negative relationship between foreign language anxiety and academic performance, indicating that higher levels of classroom anxiety are associated with lower language proficiency and poorer learning outcomes (Chen, 2025; Özdemir and Seçkin, 2025).

FLA is particularly pronounced among Chinese students, who make up the largest group of second language learners globally (Shao et al., 2013; Liu and Jackson, 2008). Various strategies have been explored to alleviate FLA, including relaxation techniques, deep breathing, and synchronous computer-mediated communication (SCMC) (Toyama and Yamazaki, 2021).

### 2.2 The use of SCMC to alleviate FLA

Computer-mediated communication (CMC) can be classified into asynchronous (ACMC) and synchronous (SCMC) forms. While ACMC involves delayed interactions (e.g., forums, emails), SCMC enables real-time communication using tools such as Zoom, Skype, and Webex, facilitating both verbal and nonverbal exchanges. Research indicates that SCMC can effectively reduce FLA (Tudini, 2007), with text-based SCMC particularly alleviating pronunciation anxiety (Satar and Özdener, 2008). Recent studies have further highlighted the affective benefits of computer-mediated interaction. Kobayashi (2025) reported that both synchronous and asynchronous CMC enhanced learners’ comfort, enjoyment, and positive emotions during online communication, while Gulzar and Jami (2022) found that SCMC helped reduce undergraduate learners’ anxiety by facilitating sustained interaction and negotiation strategies. Additionally, immersive technologies like virtual reality (VR) integrated into hybrid SCMC models further contribute to reducing FLA (York et al., 2021).

Hybrid SCMC modes—such as virtual world SCMC, VR-based SCMC, and SCMC in face-to-face (FTF) classrooms—combine the benefits of in-person interactions with computer-mediated modalities. Studies suggest that communication in virtual worlds (VWs) offers emotional benefits, such as increased willingness to communicate and reduced pressure (Reinders and Wattana, 2014). The use of avatars in VWs has been associated with lower anxiety and greater relaxation, thus enhancing students’ willingness to engage (Zhao and Lai, 2009). York et al. (2021) found that VR SCMC was the most effective in reducing FLA compared to video and voice SCMC. Liu (2023) and Xiangming et al. (2020) have shown that hybrid SCMC approaches in FTF classrooms significantly reduce FLA. Liu (2023) further highlighted that hybrid SCMC offers a better overall learning experience than pure SCMC or traditional FTF communication.

### 2.3 The advantages and disadvantages of online live teaching

Online live teaching, or pure SCMC, proved to be beneficial during the COVID-19 pandemic, particularly in alleviating FLA for shy students by offering a less intimidating platform for participation (Zhang, 2021). This mode of instruction promotes greater student engagement and self-expression through real-time interaction and feedback (Liu, 2020; Wei, 2020). Nevertheless, it is not without drawbacks. Issues such as maintaining classroom discipline and reducing digital distractions pose significant challenges, often undermining the overall effectiveness of online live teaching (Fu, 2020; Jin, 2020; Qu et al., 2021; Feng, 2020).

### 2.4 Preliminary studies on hybrid SCMC models

Recent studies suggest that hybrid SCMC models, which integrate online and face-to-face (FTF) interaction, can improve students’ psychological experiences in classroom communication and lead to a more positive overall learning experience compared with pure SCMC (Liu, 2023). Although these models do not completely eliminate internet-related distraction, they have been shown to reduce student shyness and support classroom interaction dynamics.

Building on this line of research, the integration of mobile device management (MDM) technology has been proposed as a way to regulate mobile-mediated environments in hybrid SCMC contexts (Liu, 2023). By limiting access to non-task-related applications, MDM can reduce attentional interference during class, thereby supporting classroom focus and student engagement. This perspective highlights the potential of MDM to complement hybrid SCMC by addressing distraction-related psychological challenges and underscores the need for further empirical investigation.

### 2.5 Psychological implications of mobile technology in education

The integration of mobile technology has significantly reshaped educational practices by influencing how students attend to learning tasks, interact with teachers, and experience classroom activities. Effective adoption of mobile learning (M-learning) depends on several learner-related and contextual factors, including perceived usefulness, facilitating conditions, and computer self-efficacy (Özdoğan et al., 2012). In addition, infrastructure quality and accessible content are important for supporting student engagement and learning experience in technology-rich environments (Gan and Balakrishnan, 2018; Bray, 2018; Al-Rahmi et al., 2022).

Among these factors, information quality is particularly critical, as it directly shapes learners’ perceptions of clarity, reliability, and relevance during learning activities. However, maintaining consistent information quality remains challenging in Bring Your Own Device (BYOD) environments, where device variability may increase cognitive load and attentional distraction. This situation highlights the need for centralized management approaches that support focused engagement while reducing distraction in mobile-mediated learning contexts.

### 2.6 Psychological challenges of integrating mobile technology

Although mobile technology offers considerable benefits, its integration into education also presents notable psychological challenges, including insufficient information and communication technology (ICT) support and differing levels of technology acceptance (Hyndman, 2018). The Bring Your Own Device (BYOD) approach, while advantageous for leveraging students’ familiar devices, increases exposure to non-educational content and heightens the risk of attentional distraction (Lodge, 2013; Hyndman, 2018). Recent empirical evidence suggests that such digital distractions remain prevalent in contemporary classrooms, with students reporting in-class distractions and loss of attention (Thapa et al., 2025). Research indicates that more than 80% of students use their devices for unrelated purposes, which negatively affects attention and academic performance (Lodge, 2013).

Such distractions disrupt sustained focus, reduce the effectiveness of note-taking, and are associated with poorer exam performance (Sophonhiranrak, 2021). Attah et al. (2025) further indicate that while mobile devices can enhance engagement and access to learning resources, they also contribute to cognitive overload and off-task behavior, underscoring the importance of effective device management strategies in educational settings. As a result, educators face the challenge of balancing the psychological benefits of technology-supported learning with the need to manage distraction in order to maintain a productive learning environment (Flanigan et al., 2023). Beyond distraction, BYOD strategies also raise concerns related to data security, which may further influence learners’ perceptions of technology use and underscore the need for robust policies to support responsible and focused engagement (Emery, 2012). These challenges highlight the importance of comprehensive approaches to mitigating the risks of mobile integration and point to the potential role of Mobile Device Management (MDM) technology.

### 2.7 Psychological functions of mobile device management in educational environments

Mobile device management (MDM) technology provides a structured means of regulating mobile device use within educational environments. Although originally developed to enhance device security, MDM systems have expanded to address broader educational contexts by shaping how learners interact with mobile media, including application management, content filtering, and remote device control. These functions are particularly relevant in BYOD settings, where variation in student-owned devices increases the need for consistent regulation (GoGuardian, 2021; Zimmerman, 2019); from a psychological perspective, such regulation may help reduce distraction and support focused engagement. From a pedagogical perspective, recent research suggests that teachers may perceive managed uses of mobile technology as supportive of instructional innovation and classroom organization, highlighting the potential role of device regulation in technology-enhanced teaching contexts (Burke et al., 2025).

By enabling the regulation of device access and usage during class, MDM reduces exposure to non-task-related content and supports more stable attentional focus. In this way, it contributes to classroom organization, facilitates collaborative learning activities, and limits psychologically distracting stimuli rather than merely providing technical oversight (Saltmarsh, 2014).

### 2.8 Psychological rationale for MDM in educational contexts

The increasing reliance on mobile devices in K–12 education, driven by one-to-one initiatives and BYOD programs, highlights the growing psychological demands placed on learners in mobile-mediated classrooms. Martin et al. (2025) systematically reviewed the literature on digital distractions in educational settings and found that technology distractors, personal needs, and instructional environment factors jointly contribute to attentional interference, with prevention strategies including classroom regulations and technology controls. Effective MDM solutions help structure learning environments by regulating application access, managing device use, and enforcing security measures. By limiting exposure to non-task-related content and safeguarding sensitive data, MDM supports focused engagement and stable learning experiences in technology-rich settings (Zimmerman, 2019; Arden, 2023; GoGuardian, 2021).

As the adoption of BYOD strategies expands, managing diverse personal devices becomes increasingly complex and may intensify distraction and attentional fragmentation. In this context, MDM systems play an important role in reducing distraction and supporting sustained attention, enabling mobile technology to function as a facilitator of learning rather than a source of psychological disruption.

### 2.9 Empirical evidence and psychological implications of MDM

Empirical evidence from school districts such as the Mooresville Graded School District (MGSD) and the Northborough–Southborough public schools illustrates how MDM implementation has been associated with changes in learning environments supported by digital technology. The MGSD in Mooresville, North Carolina, implemented a 1:1 laptop initiative beginning in 2008, which substantially altered classroom practices. According to Scott Smith, Chief Technology Officer at MGSD, the district’s “digital conversion” was accompanied by improved academic achievement, reduced dropout rates, and increased graduation rates, with MDM tools such as FileWave supporting consistent device access and individualized software availability (Smith, 2016).

In Northborough–Southborough, Massachusetts, the adoption of the MDM solution Light speed similarly influenced the organization of technology-supported classrooms. Leo Brehm, Director of Technology and Digital Learning, noted that centralized MDM tools streamlined updates and device access while providing teachers with greater flexibility in managing classroom devices, contributing to a more structured and responsive learning environment (Smith, 2016).

While these cases offer informative contextual examples, they are largely based on school-level outcomes and professional reflections rather than direct psychological measurement. This highlights the need for systematic empirical research to examine how MDM shapes learners’ attentional, emotional, and experiential responses within technology-mediated educational settings.

### 2.10 Integrating MDM into hybrid SCMC: research gaps and hypothesis

Taken together, existing studies provide a comprehensive understanding of both the affordances and constraints of technology-supported learning. Research on foreign language anxiety (FLA) has consistently demonstrated its negative relationship with learning outcomes, while studies on synchronous and hybrid SCMC have shown that mediated interaction can reduce anxiety and foster a more supportive classroom climate (Tudini, 2007; York et al., 2021; Kobayashi, 2025). At the same time, research on online live teaching and mobile-supported instruction has repeatedly identified digital distraction and attentional fragmentation as persistent challenges in technology-rich and BYOD classroom environments (Lodge, 2013; Thapa et al., 2025).

Parallel work on mobile technology use further indicates that, although mobile devices can enhance engagement and access to learning resources, unrestricted device use often increases cognitive load and off-task behavior, thereby undermining instructional effectiveness (Attah et al., 2025; Martin et al., 2025). Collectively, these findings suggest that the effectiveness of technology-enhanced instruction depends not only on communicative affordances, but also on the attentional conditions under which such affordances are realized.

Despite these advances, several gaps remain in the literature. First, although hybrid SCMC has been examined in authentic classroom settings and shown to improve learners’ affective experiences (Liu, 2023), internet-related distraction has often been acknowledged as a contextual limitation rather than systematically addressed through instructional design or technological regulation. As a result, the conditions under which the affective benefits of hybrid SCMC can be sustained in mobile-supported classrooms remain insufficiently examined.

Second, while attentional regulation and digital distraction have been widely studied across diverse educational contexts, these perspectives have rarely been empirically integrated into communication-centered learning environments such as hybrid SCMC. Third, research on Mobile Device Management (MDM) in education has primarily focused on technical functions, policy considerations, or descriptive practitioner-level implementation reports, with limited learner-level empirical evidence examining its psychological effects on attention and learning experience (Emery, 2012; Samochadin et al., 2015; Smith, 2016).

Addressing these gaps, the present study integrates Mobile Device Management (MDM) into a hybrid SCMC classroom context to examine how attentional regulation shapes learners’ experiences in mobile-supported instruction. Building on previous findings regarding the affective benefits of hybrid SCMC, this study hypothesizes that integrating Mobile Device Management (MDM) into hybrid SCMC will reduce internet-related distraction and lead to more favorable learning outcomes in mobile-supported classrooms.

## 3 Materials and methods

### 3.1 Research design and methodology

A self-contrast study was conducted to investigate a single group of 60 students who participated in three learning modes: the normal classroom mode (control group), the hybrid SCMC (no MDM) mode, and the hybrid SCMC (with MDM) mode (test groups). This methodology allowed participants to compare their experiences across the three modes directly, thus providing clear insights into the differences in their foreign language anxiety (FLA) and perceptual experiences. Students’ FLA scores and experiences were assessed via questionnaires. Pretest surveys were not conducted in this self-contrast study to prevent practice effects resulting from repetitive testing. The normal classroom mode served as the control group, thus establishing a baseline for comparison and providing a blank sample prior to the treatment conditions.

### 3.2 Observed learning activity

This study focused on a mandatory English course for second-year Chinese students who were not English majors. This course, which includes reading, listening, and translation exercises, is a common requirement at nearly all Chinese universities, thus making it representative of the broader context of second language learning in China. Given that the second language acquisition population in China is among the largest worldwide, this course provides a significant foundation for understanding the challenges and dynamics associated with English language learning in this setting. In this study, all 60 students participated in courses across all three modes, in which context each mode consisted of eight 45-min sessions. Teachers initiated a specific number of interactions in each class and waited approximately 60 seconds for student responses after each question was asked.

### 3.3 Normal classroom mode

In the normal classroom mode, students engage in traditional face-to-face communication without using any online teaching platform technologies. They all have smartphones at their hand, so they may use their personal smartphones to access the internet during the course. This mode serves as a control group, thereby providing a baseline for comparison with other modes; this approach thus represents typical teaching scenarios in daily practice.

### 3.4 Hybrid SCMC (no MDM) mode

**FIGURE 1:**
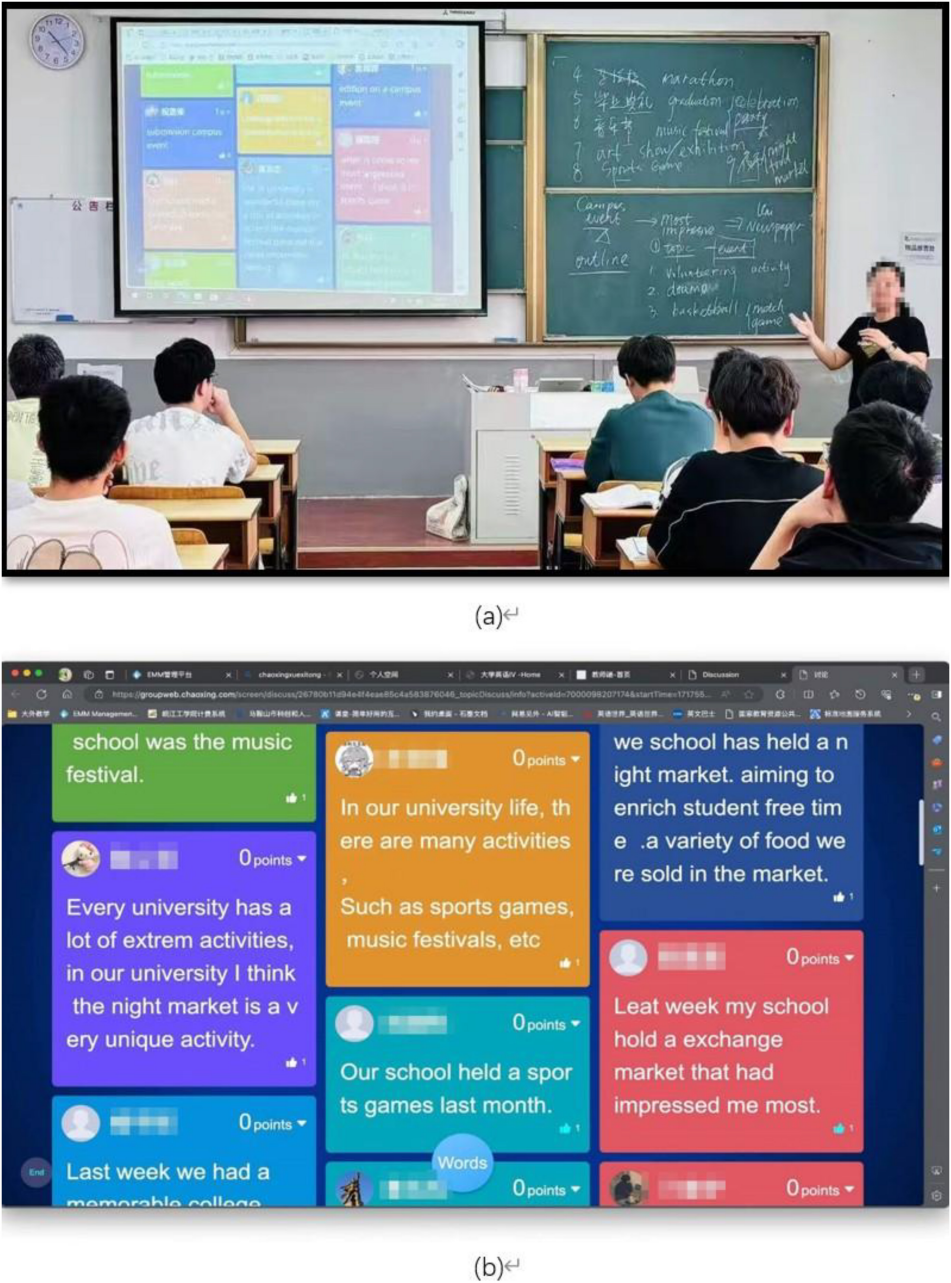
Classroom interactions through hybrid synchronous computer-mediated communication (SCMC). **(a)** Students participate in classroom activities using either face-to-face communication or text-based SCMC under hybrid modes with or without mobile device management (MDM). **(b)** The projected SCMC interface illustrates examples of students’ text-based interactions. The content shown here is not the real-time screen captured at the moment of the classroom photo in **(a)**, but an authentic screenshot of the SCMC posts generated during the same activity.

In the hybrid SCMC (no MDM) mode, students are located in a face-to-face classroom while simultaneously participating in online interactive sessions via their smartphones with open internet access. This approach allows students to interact via either text-based SCMC or traditional face-to-face methods (see Figure 1).

### 3.5 Hybrid SCMC (with MDM) mode

In the hybrid SCMC (MDM) mode, students learn in a face-to-face classroom while participating in online interactive sessions via their smartphones, in which context their internet access is controlled via MDM. This setup allows students to interact via either text-based SCMC or traditional face-to-face methods (see Figure 1).

### 3.6 SCMC software: superstar learn version 6.33

#### 3.6.1 Implementation methods

**FIGURE 2:**
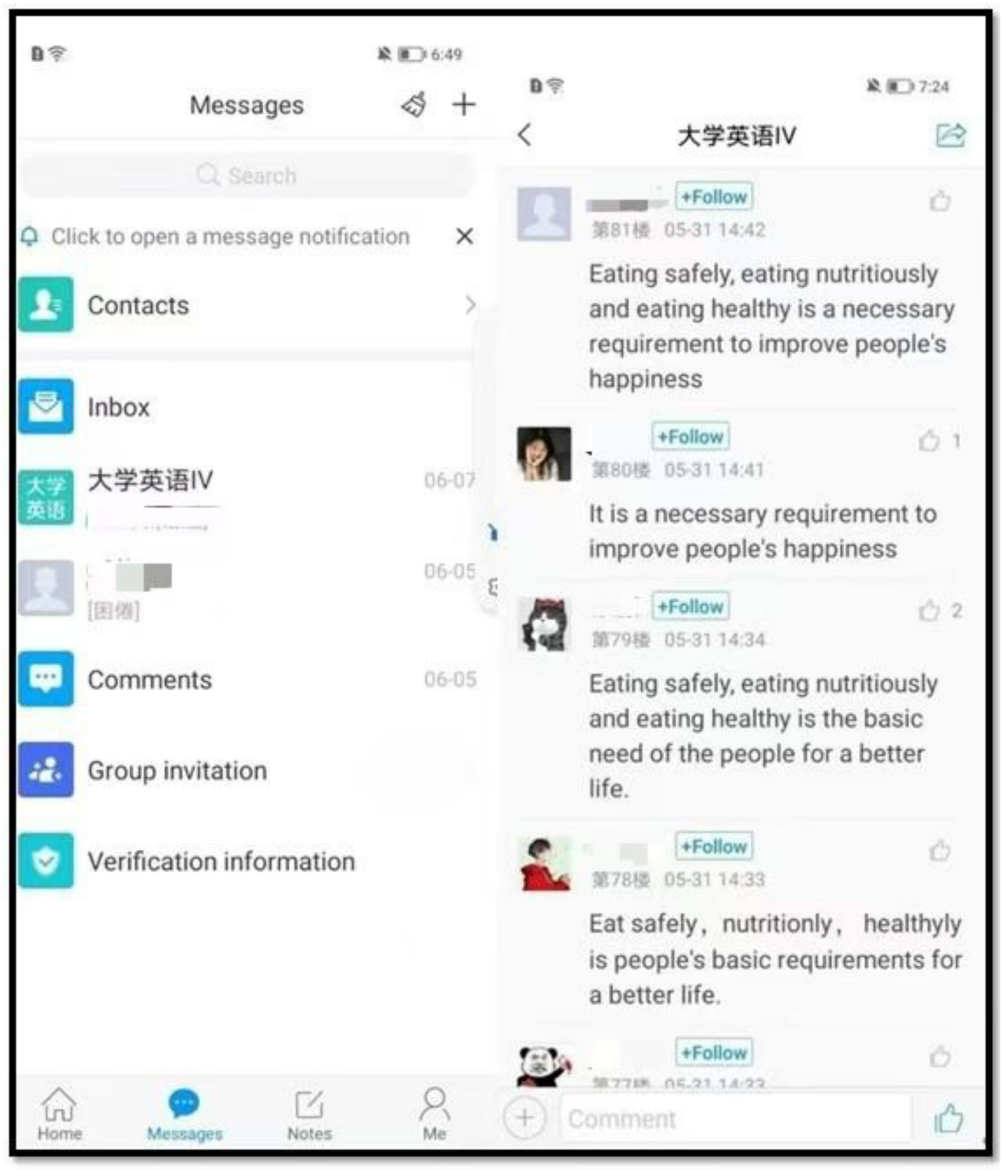
The chat room module interface of Superstar Learn, which students accessed via their mobile phones.

Students primarily use the chat room module of the platform to interact via text-based synchronous computer-mediated communication (SCMC) on their mobile phones (see Figure 2). This chat room functions in a manner similar to many common chat applications, such as Skype, including by allowing users to send text messages, images, and voice recordings. In this study, we primarily utilized the text chat feature to establish a space in which students could respond and communicate during class, thereby facilitating real-time interaction and engagement in the learning process.

### 3.7 MDM Software and control principles

**FIGURE 3:**
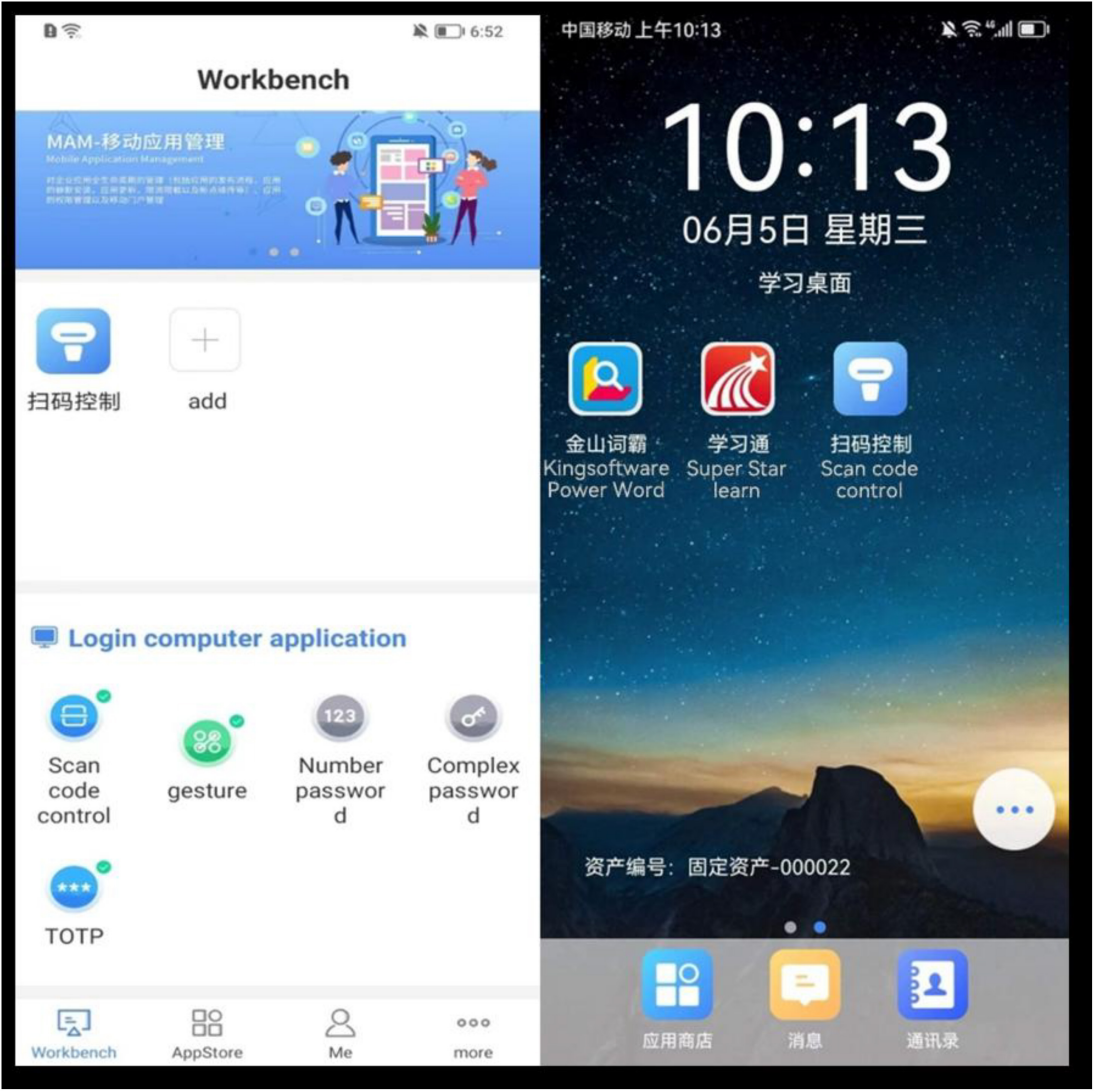
The scan-in or log-in interface presented to students on their smartphones and a locked desktop on which only two types of learning software are available.

- *Software Name*: UniEMM Enterprise Mobile Security Management Platform
- *Version:* V5.0
- This software was chosen because it is one of the major MDM solutions currently used in China and is employed by various governmental entities, such as the Bank of China, China Insurance, and state-run hospitals. While no MDM software has been specifically designed for educational environments in China, UniEMM is a frequently used platform that provides strong mobile security management features. Its established presence in the market made it a suitable choice for this research, thus allowing us to take advantage of familiar technology to address the challenges associated with managing mobile devices in an educational context.
- *Control principles:* This MDM software supports the centralized management of mobile devices by allowing administrators to customize security policies, including application whitelists, operating system version restrictions, subscriber identity module (SIM) card change policies, and feature restrictions (e.g., disabling cameras, screenshots, and app installations). The main strategy employed in this context involves locking desktops (see Figure 3) and using application whitelists, which include only approved educational apps such as Superstar Learn and Kingsoft Power Word (Dictionary), thereby ensuring that students cannot access nonlearning-related content during class.

### 3.8 MDM implementation methods

- *BYOD (Bring Your Own Device):* During class, participants scan a code or manually activate the MDM software, which then controls their personal smartphones. The locked desktop allows only the use of approved educational apps. After class, teachers manually deactivate the control mode.
- *COPE (Corporate-Owned, Personally Enabled):* Due to issues related to mobile phone permissions and licenses regarding the use of MDM software in China, UniEMM has traditionally been implemented in corporate environments by rooting phones or using COPE methods. In this study, we sought to avoid modifying participants’ personal devices, which is why we provided school-owned devices to 45 of the 60 participants whose phones could not be fully integrated with the UniEMM platform without modification. These COPE devices, which were equipped with the same MDM and SCMC platforms, allowed us to simulate a BYOD environment without the need to alter students’ personal devices. Although MDM continues to exhibit certain permission-related limitations in China, these technical barriers are expected to be removed as the value of MDM technologies becomes increasingly recognized by mobile manufacturers.

**FIGURE 4:**
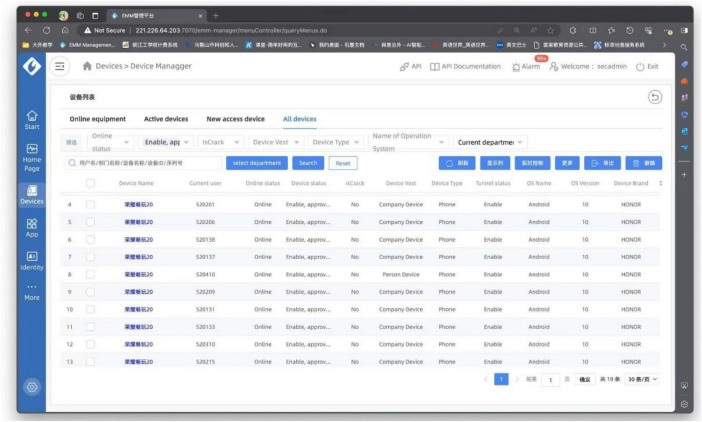
The MDM control interface for teachers.

The MDM control interface for teachers (See Figure 4) displays all logged-in devices; each line represents a different device. By clicking the boxes in the left column and selecting the deactivate option, teachers can release MDM control from specific devices.

### 3.9 Foreign Language Classroom Anxiety Scale

After the students completed the learning activities in each mode, they were required to complete the FLCAS questionnaire for each mode with the goal of measuring their foreign language learning anxiety (see Supplementary Appendix 1). This study focused on 20 items pertaining to classroom interaction that were drawn from the 33-item FLCAS questionnaire; the aim of this process was to observe students’ anxiety concerning classroom interaction. The validity and reliability of the questionnaire met the requirements for the experiments, as indicated by a Cronbach’s alpha coefficient of 0.933; furthermore, the high level of validity exhibited by the questionnaire was demonstrated through factor analysis.

### 3.10 Perception questionnaires

After completing all three instructional modes, students responded to a set of perception questionnaires designed to assess four dimensions based on learners’ subjective evaluations: learning anxiety, smartphone internet-related distraction, classroom atmosphere, and overall learning experience (see Supplementary Appendices 2–5). Each dimension was measured separately for the three instructional modes.

The perception questionnaires were self-designed by the research team based on the specific aims of the study and prior research on affective and perceptual factors in technology-mediated language learning. The development process followed an iterative and structured procedure. First, the researchers identified the key constructs and drafted an initial pool of items for each dimension. AI tools were used at this stage solely to assist with wording exploration and coverage checking. All items were subsequently reviewed, discussed, and refined by the research team, who have professional backgrounds in language education and educational technology, to ensure theoretical relevance, clarity, and content representativeness.

Before formal implementation, the questionnaires were pilot-tested with 40 non-experimental students from the same university who were not involved in the main study. Based on feedback from the pilot participants, minor wording revisions were made to improve item clarity and consistency. The revised versions were subsequently subjected to reliability and exploratory factor analyses. The results demonstrated high internal consistency across all subscales, with Cronbach’s alpha coefficients of 0.957 for anxiety, 0.949 for classroom atmosphere, 0.973 for overall learning experience, and 0.888 for smartphone internet-related distraction.

Exploratory factor analyses further supported the internal structure of the questionnaires. For the anxiety scale, the Kaiser–Meyer–Olkin (KMO) value was 0.869, and Bartlett’s test of sphericity was significant (χ^2^ = 331.762, df = 28), indicating suitability for factor analysis. Similar results were obtained for classroom atmosphere (KMO = 0.905, Bartlett’s χ^2^ = 310.510), overall learning experience (KMO = 0.838, Bartlett’s χ^2^ = 455.347), and smartphone internet-related distraction (KMO = 0.867, Bartlett’s χ^2^ = 210.109). In each case, factor extraction revealed clear and theoretically interpretable structures with satisfactory item loadings, providing preliminary evidence for the structural coherence of the instruments.

Based on the pilot results, the questionnaires were considered appropriate for use in the main experiment. To further examine measurement stability, reliability indices and factor structures were re-evaluated across the three instructional conditions in the main study. This approach allowed the consistency of questionnaire performance to be monitored under different instructional contexts and ensured that the measurement results were not dependent on a single testing occasion.

### 3.11 Open-ended responses

At the end of each perception questionnaire regarding anxiety, the classroom atmosphere, and distraction resulting from the mobile internet, the students provided one open-ended response. They were asked to explain their ratings in one or two sentences with the goal of helping the researchers understand the reasons and feelings underlying their scores.

### 3.12 Study subjects

This study involved 60 second-year undergraduate students (M age = 20.6, SD = 0.743) who were non-English majors and were recruited from a university in MaAnShan, People’s Republic of China. The sample included 24 females and 36 males, all of whom had successfully passed the national college entrance examination and were enrolled in a 4-year full-time undergraduate program. These students were enrolled in a mandatory English course designed for second-year students who were not majoring in English. This course is a public compulsory course that is required for nearly all non-English major students in Chinese universities, and as such, it represents a typical environment for college foreign language learning in China.

The students were admitted to the university under the second batch of undergraduate admissions, which represents the lower part of the undergraduate admission spectrum in China. This places them at an overall academic level that is generally considered middle to lower within the range of Chinese universities and colleges, which makes them representative of the foreign language learning situation for Chinese university students at the median level, or the largest group of students in terms of numbers. Despite the fact that these students had completed 11 years of compulsory English education, their overall levels of English proficiency remained relatively low. Specifically, only 25% of the students had passed the College English Test 4 (CET4), a national standardized test in China that requires a minimum passing score of 425, by the end of their second year.

Participation in this study was voluntary. Students were selected based on the order of application and the compatibility of their smartphones with the UniEMM software. For students whose personal devices were incompatible with the MDM system, COPE smartphones were temporarily provided to ensure that all participants could access the MDM-controlled learning environment.

### 3.13 Study indicators

This study used the FLCAS, perception questionnaires, and open-ended responses to produce a comprehensive evaluation of learners’ psychological experiences across different media-mediated conditions.

### 3.14 Procedure

**FIGURE 5:**
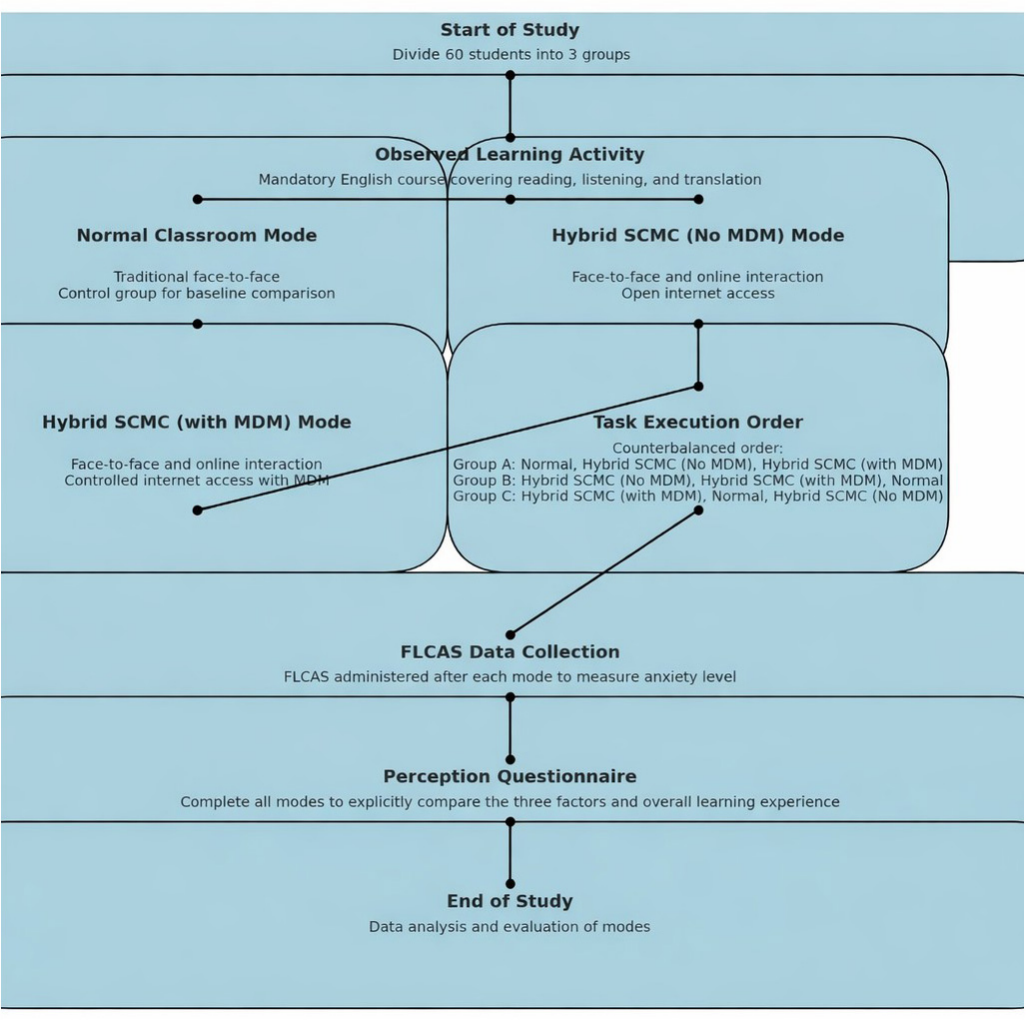
Research procedure.

The 60 students were divided into 3 groups and experienced the three modes in a 3 × 3 counterbalanced order with the goal of eliminating any possible sequence effects. After the students progressed through each mode, they completed the FLCAS questionnaire. Given the large number of items included in this questionnaire, the questionnaire was administered after each mode with the goal of capturing students’ immediate levels of anxiety. After the students completed all the modes, they responded to four sets of perception questionnaires, which focused on anxiety, the classroom atmosphere, distractions resulting from the mobile internet, and the overall learning experience. Each perception questionnaire contained approximately 10 questions per dimension with the goal of facilitating explicit comparisons among the three modes. The fact that the comparison was conducted after all modes had been completed offered clearer insights into the study’s primary focus, i.e., the comparative advantages and disadvantages associated with each mode. At the end of each perception questionnaire, students were asked to answer an open-ended question to explain the reasons underlying their choices regarding the three factors in the perception questionnaire (see Figure 5).

### 3.15 Statistical analysis and AI assistance

Statistical analyses were performed with Stata 18 (StataCorp LLC, College Station, TX, United States). Normality was evaluated using the Shapiro–Wilk test, followed by a one-way repeated-measures ANOVA; when the normality assumption was violated, the Wilcoxon signed-rank test was applied. The Friedman test and Kendall’s coefficient of concordance (W) were calculated with the web-based package StatsToDo.^1^ Initial questionnaire wording was developed with the assistance of a large language model (ChatGPT, OpenAI) for wording exploration. AI tools were also used to assist with figure preparation. Figure 1 is a schematic illustration of the study procedure. All other figures presenting results were generated from the analyzed dataset (data-to-figure visualization) and were verified by the authors against the raw data. Draft text was refined for language clarity. All content was reviewed, revised, and approved by the authors, who take full responsibility for the manuscript.

## 4 Results

To examine how mobile device management (MDM) and hybrid synchronous computer-mediated communication (SCMC) shape learners’ psychological experiences, a self-contrast approach was employed to compare students’ responses across three media-mediated conditions. These conditions corresponded to a normal classroom environment, a hybrid SCMC environment without MDM, and a hybrid SCMC environment with MDM. This design allowed for direct comparison of students’ foreign language anxiety (FLA) and learning experience under different levels of media regulation. The results presented below illustrate how the integration of MDM with SCMC influences students’ engagement, anxiety, and perceived interaction context.

### 4.1 Analysis of the FLCAS results

Based on responses from 60 participants, the FLCAS was administered under three instructional modes to assess differences in foreign language anxiety (see Supplementary Appendix 1). Table 1 presents the results of the normality tests, repeated-measures ANOVA, and Bonferroni-adjusted *post-hoc* comparisons.

**TABLE 1:** Normality test, repeated measures ANOVA, and *post-hoc* comparisons of FLCAS scores (*N* = 60).

Normality Test (Shapiro–Wilk)				
Mode		Shapiro–Wilk statistic	*p*-value	
Normal mode		0.9831	0.5715	
Hybrid SCMC (no MDM)		0.9700	0.1461	
Hybrid SCMC (with MDM)		0.9850	0.6680	
**Repeated measures ANOVA**				
**Source**	**df**	***F***	***p***	**partial η^2^**
Mode	2,118	16.20	< 0.001	0.22
**Post-hoc Comparisons of Marginal Means (Bonferroni-adjusted α≈ 0.0167)**				
**Comparison (A vs. B)**		**Mean difference (A–B)**	**95% CI**	***p***
Normal vs. Hybrid SCMC (no MDM)		6.150	2.80–9.50	0.0000
Normal vs. Hybrid SCMC (with MDM)		7.317	3.96–10.67	0.0000
Hybrid SCMC (no MDM) vs. Hybrid SCMC (with MDM)		1.167	−2.19 to 4.52	0.4000

Huynh–Feldt correction applied (ε = 0.93).

#### 4.1.1 Normality test

All three instructional modes met the normality assumption (*p* = 0.5715, 0.1461, and 0.6680), allowing the use of repeated measures ANOVA.

#### 4.1.2 RM ANOVA

The omnibus test revealed a significant main effect of instructional mode on FLCAS scores, *F*(2,118) = 16.20, *p* < .001, with a moderate effect size (partial η^2^ =.22).

*Post-hoc* pairwise comparisons (Bonferroni-adjusted).

- Normal vs. hybrid SCMC (no MDM): *p* = 0.0000, which is smaller than the Bonferroni-adjusted α = 0.0167; the difference is therefore significant.
- Normal vs. hybrid SCMC (with MDM): *p* = 0.0000, also below α = 0.0167; the difference is significant.
- Hybrid SCMC (no MDM) vs. hybrid SCMC (with MDM): *p* = 0.4000, which exceeds α = 0.0167; the difference is not significant.
- To control the family-wise Type I error rate for the three pairwise comparisons, a Bonferroni correction was applied, lowering the significance threshold from 0.05 to 0.05 / 3 ≈ 0.0167.

**FIGURE 6:**
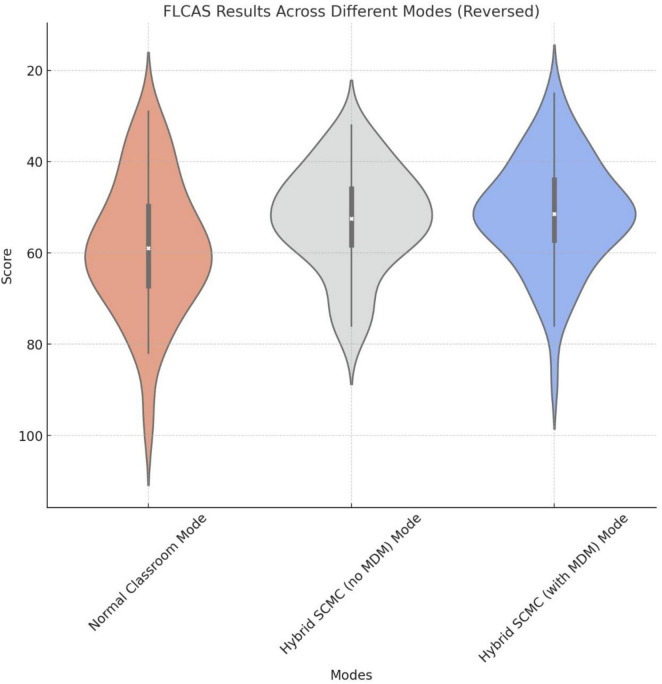
Graphical representation of the FLCAS results (Axis is reversed since FLCAS is a negative factor).

In this study, the original FLCAS scores were retained for all statistical analyses. Because foreign language anxiety represents a negative affective construct (i.e., higher scores indicate higher anxiety), the vertical axis in Figure 6 was visually inverted to facilitate intuitive interpretation of the results.

This axis inversion allows lower anxiety levels to appear higher in the graphical display, aligning the visual direction of the figures with the pedagogical interpretation of improved learning conditions as a more positive outcome. It is important to note that this was solely a visualization adjustment; no reverse scoring or numerical transformation of the raw questionnaire data was conducted.

These results indicate that both hybrid SCMC modes (i.e., the modes both with and without MDM technology), significantly reduce students’ foreign language learning anxiety. The differences between the two hybrid SCMC modes are not significant, thus suggesting that MDM technology has a minor effect on this dimension (Figure 6 and Table 1).

The findings imply that incorporating the hybrid SCMC mode into the classroom can effectively reduce foreign language anxiety, regardless of whether MDM technology is used. This suggests that the benefits of SCMC in enhancing student engagement and interaction may play a more prominent role in reducing anxiety than the distraction control afforded by MDM. Future research should further explore the specific contributions of SCMC and MDM to ensure a balanced understanding of their roles in reducing student anxiety.

### 4.2 Responses to the anxiety perception questionnaire

The Anxiety Perception Questionnaire was used to complement the FLCAS and to enable comparative evaluation of foreign language anxiety (FLA) after participants experienced all three instructional modes (see Supplementary Appendix 2). To assess measurement stability, reliability and factor structure were examined separately for each mode based on responses from 60 participants. The questionnaire showed consistently high internal consistency, with Cronbach’s alpha values of 0.942 (normal), 0.946 (no-MDM), and 0.940 (with-MDM). Exploratory factor analyses further supported structural coherence, with KMO values ranging from 0.877 to 0.932 and significant Bartlett’s tests in all cases. These results indicate stable reliability and structural consistency across instructional conditions, supporting the validity of cross-mode comparisons.

#### 4.2.1 Quantitative analysis of anxiety perception questionnaire results

Quantitative responses were analyzed using appropriate non-parametric procedures to examine differences in anxiety perception across instructional modes. Friedman tests and *post-hoc* Wilcoxon signed-rank comparisons were conducted to assess overall and pairwise differences. The results indicate significant reductions in perceived anxiety in both hybrid SCMC modes relative to the normal classroom mode.

##### 4.2.1.1 Normality test

The normal mode met the normality assumption (*p* = 0.2782), whereas both hybrid SCMC modes deviated from normality (*p* = 0.0470 and 0.0463), requiring non-parametric analyses.

##### 4.2.1.2 Friedman test

The Friedman test revealed a significant overall difference in anxiety-perception scores across the three instructional modes, χ^2^(2) = 62.774, *p* < 0.0001, with a large effect size (Kendall’s *W* = 0.664).

##### 4.2.1.3 *Post-hoc* comparisons

Pairwise comparisons were conducted using a Bonferroni-corrected significance threshold (α≈ 0.0167).

- Normal vs. Hybrid SCMC (no MDM): significant (*p* = 0.0000 < 0.0167).
- Normal vs. Hybrid SCMC (with MDM): significant (*p* = 0.0000 < 0.0167).
- Hybrid SCMC (no MDM) vs. Hybrid SCMC (with MDM): not significant (*p* = 0.1022 > 0.0167).

**FIGURE 7:**
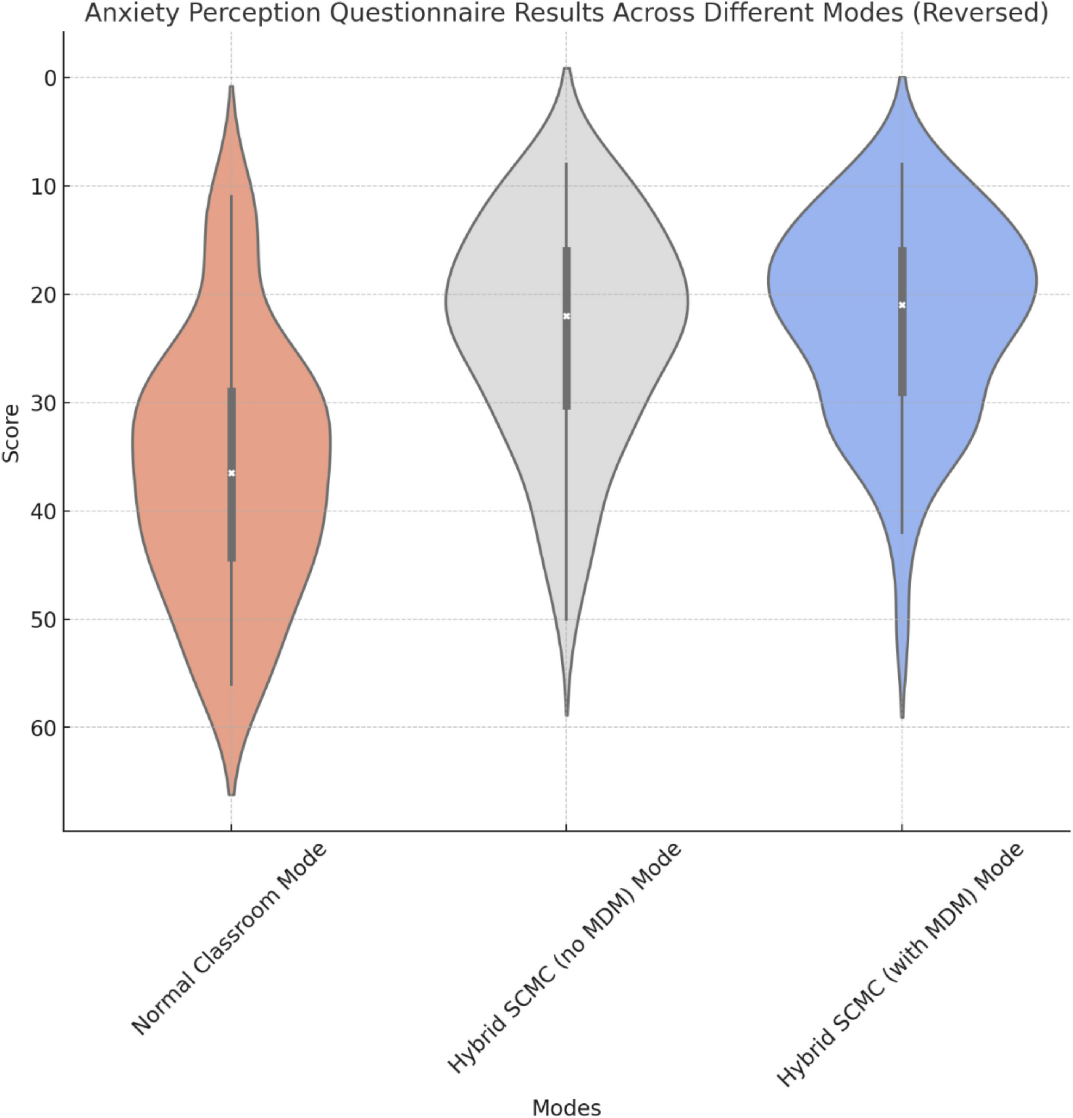
Graphical representation of responses to the Anxiety Perception Questionnaire (Axis is reversed since anxiety is a negative factor with regard to learning experience).

These results of this research highlight significant differences in students’ anxiety perception scores between the normal and hybrid SCMC modes, in which context the hybrid SCMC modes significantly reduced students’ anxiety. However, the differences between the two hybrid SCMC modes were not significant, which is in line with the results of the FLCAS. The violin plot comparison including FLCAS indicates a consistent trend: both SCMC modes resulted in lower levels of anxiety than did the normal classroom mode (baseline), and a similar degree of reduction was observed in both modes. However, the results of the perception questionnaire revealed a more pronounced difference between the SCMC modes and the baseline mode (Figure 7 and Table 2).

**TABLE 2:** Normality test, Friedman test, and Pairwise Wilcoxon Signed-Rank comparisons of anxiety-perception scores (*N* = 60).

Normality Test (Shapiro–Wilk)			
Mode	Shapiro–Wilk Statistic		*p*-value
Normal	0.9758		0.2782
Hybrid SCMC (no MDM)	0.9600		0.0470
Hybrid SCMC (with MDM)	0.9599		0.0463
**Friedman test**			
**Test statistic**	**df**	**Kendall’s W**	***p*-value**
χ^2^(Friedman) = 62.774	2	0.664	<0.0001
**Pairwise Wilcoxon Signed-Rank Tests (Bonferroni-adjusted α ≈ 0.0167)**			
**Comparison (A vs. B)**	***z***	**p (two-tailed)**	**Mean difference (A–B)**
Normal vs. Hybrid SCMC (no MDM)	6.447	0.0000	12.7167
Normal vs. Hybrid SCMC (with MDM)	6.108	0.0000	13.8667
Hybrid SCMC (no MDM) vs. Hybrid SCMC (with MDM)	1.634	0.1022	1.1500

This finding may be explained in two ways:

First, although the FLCAS questionnaire included 20 items related to classroom interaction, derived from the original 33 questions, some of these items were not closely linked with interaction specifically. In contrast, the Anxiety Perception Questionnaire focused exclusively on anxiety related to expressions and interactions in the classroom, thus providing more targeted feedback (see Supplementary Appendix 2). This finding aligns with the hypothesis of previous research that SCMC modes primarily reduce anxiety associated with classroom expressions and interactions, thereby increasing participants’ willingness to engage. It also suggests that SCMC modes may not significantly alleviate other aspects of foreign language learning anxiety unrelated to direct classroom interactions.

Second, the Anxiety Perception Questionnaire was administered after the students had completed all three modes and was designed to facilitate explicit comparisons among the three modes. The stronger contrast observed in the results is in line with the researchers’ original expectations regarding the test.

#### 4.2.2 Qualitative analysis of open-ended responses to the anxiety perception questionnaire

A total of 50 valid responses were collected from the open-ended section of the Anxiety Perception Questionnaire. These textual responses were analyzed using a qualitative content analysis approach. Following an inductive procedure, responses were first subjected to open coding, after which conceptually similar codes were iteratively grouped into broader thematic categories. As individual responses often addressed multiple aspects of anxiety experience, overlapping codes were permitted. AI tools were used to assist with the organization and retrieval of textual data, while all coding decisions and thematic refinement were conducted by the research team. The analytic procedure was consistent with that applied in other open-ended sections of the study.

Through iterative coding, seven recurring codes were identified and organized into three higher-order themes, reflecting dominant patterns as well as contrasting individual experiences related to anxiety across instructional modes.

Theme 1: Anxiety Reduction through Mediated Participation in Hybrid SCMC Modes

This dominant theme reflects students’ perceptions that hybrid SCMC environments reduce anxiety by lowering the social and performance pressure typically associated with face-to-face classroom interaction.

- *Tension in the normal classroom mode* (26 responses):
  Students frequently reported feeling nervous in traditional classrooms when answering questions, mainly due to fear of making mistakes, pronunciation concerns, or being evaluated publicly.
- *Increased classroom participation in hybrid SCMC modes* (5 responses):
  Online-mediated interaction allowed students to respond without direct face-to-face exposure, which was perceived as less threatening and more comfortable.
- *Increased thinking time in hybrid SCMC modes* (1 response):
  A small number of students noted that having additional time to think before responding helped alleviate anxiety.

Overall, these responses indicate that mediated participation and reduced social pressure constitute the primary pathways through which hybrid SCMC modes support anxiety reduction.

##### 4.2.2.1 Theme 2: focus-related experiences in the MDM mode

This theme reflects a minority of student responses reporting feeling more settled in the MDM-supported hybrid SCMC mode.

- *Focus in the MDM mode helped* (9 responses):
  Several students noted that restrictions on non-learning-related content helped them concentrate more during class and feel more settled during learning activities.

These responses were *less frequently reported* than those emphasizing reduced social pressure or increased relaxation in other modes and therefore represent *a secondary pattern* in the qualitative data.

##### 4.2.2.2 Theme 3: divergent and contrasting anxiety experiences across instructional modes

In contrast to the dominant pattern, a subset of responses expressed divergent or opposing views, particularly toward the MDM-supported hybrid SCMC mode.

- *Relaxation in the normal classroom mode* (3 responses):
  Some students reported feeling most relaxed in the traditional classroom, attributing this to the absence of technological restrictions and a greater sense of freedom.
- *Advantages of each mode* (3 responses):
  A small number of students perceived no substantial differences in anxiety across instructional modes, emphasizing individual preference.
- *Lowest anxiety in the hybrid SCMC (without MDM) mode* (5 responses):
  Several students reported experiencing the least anxiety in the hybrid SCMC mode without MDM, noting that the ability to search for information freely without restrictions reduced stress.

Notably, these contrasting views were *concentrated in the newly introduced instructional modes*, particularly those involving combined SCMC and MDM technologies.

#### 4.2.3 Interpretive considerations and quantitative alignment

The qualitative patterns correspond closely to the quantitative findings reported in sections 4.1 and 4.2.1. Both the FLCAS and the Anxiety Perception Questionnaire showed significantly higher anxiety levels in the normal classroom mode than in the two hybrid SCMC modes (*p* < .001), while no significant difference emerged between the two hybrid conditions. The distribution of open-ended responses mirrors this statistical pattern. A substantial number of responses (26 cases) explicitly described tension and fear of public evaluation in the normal classroom, aligning with the elevated anxiety scores in that condition. In contrast, several responses emphasized reduced pressure and more comfortable participation in hybrid SCMC modes, corresponding to the significant reductions observed in both quantitative instruments.

At the same time, a smaller number of contrasting responses (e.g., 3 cases favoring the normal mode and 5 cases preferring the hybrid SCMC mode without MDM) reflect minority variation rather than a systematic reversal of the overall trend. These differences may be interpreted in two complementary ways. First, they may reflect *individual variability in adapting to newly introduced technologies*, especially given the relatively short implementation period. Second, they suggest a tension between attentional control and perceived autonomy, whereby restrictions designed to reduce distraction may be experienced as limiting by some learners. These considerations help contextualize the observed heterogeneity while reinforcing the dominant pattern that anxiety reduction is primarily associated with mediated participation in hybrid SCMC modes.

#### 4.2.4 Summary

Overall, the qualitative analysis indicates that hybrid SCMC modes are generally perceived as effective in reducing foreign language learning anxiety by lowering social pressure and providing more flexible participation opportunities. At the same time, students’ open-ended responses reveal meaningful individual differences, particularly in relation to the MDM-supported mode. While some students associated increased focus with a calmer learning experience, others reported greater relaxation in less restricted environments. These findings underscore the importance of accounting for learner heterogeneity and autonomy considerations when designing technology-supported instructional modes.

### 4.3 Responses to the classroom atmosphere perception questionnaire

The Classroom Atmosphere Perception Questionnaire was administered after participants experienced all three instructional modes to enable holistic comparisons of perceived classroom environment across conditions (see Supplementary Appendix 3). To examine measurement stability, reliability and factor structure were analyzed separately for each mode based on responses from 60 participants. The questionnaire demonstrated high internal consistency across modes, with Cronbach’s alpha values of 0.945 (normal), 0.935 (no MDM), and 0.945 (with MDM). Exploratory factor analyses further supported structural coherence, with KMO values ranging from 0.908 to 0.927 and significant Bartlett’s tests in all cases (χ^2^ = 368.374–452.890). These results indicate stable reliability and structural consistency across instructional conditions, supporting valid cross-mode comparisons.

#### 4.3.1 Quantitative analysis of classroom atmosphere perception results

Quantitative responses were analyzed using appropriate non-parametric procedures to examine differences in perceived classroom atmosphere across instructional modes. Friedman tests and pairwise Wilcoxon signed-rank comparisons were applied to assess overall and pairwise differences. The results demonstrate significant improvements in classroom atmosphere ratings for both hybrid SCMC modes compared to the normal classroom mode, with additional gains observed in the MDM-supported condition.

##### 4.3.1.1 Normality test

The normal mode met the normality assumption (*p* = 0.7075), whereas both hybrid SCMC modes deviated from normality (*p* = 0.0423 and 0.0168), requiring the use of non-parametric procedures.

##### 4.3.1.2 Friedman test

The Friedman test revealed a significant overall difference in classroom-atmosphere scores across the three instructional modes, χ^2^(2) = 52.361, *p* < 0.0001, with a large effect size (Kendall’s *W* = 0.507).

##### 4.3.1.3 *Post-hoc* comparisons

Pairwise comparisons were conducted using a Bonferroni-corrected significance threshold (α≈ 0.0167).

- Normal vs. Hybrid SCMC (no MDM): significant (*p* = 0.0000 < 0.0167).
- Normal vs. Hybrid SCMC (with MDM): significant (*p* = 0.0000 < 0.0167).
- Hybrid SCMC (no MDM) vs. Hybrid SCMC (with MDM): significant (*p* = 0.0002 < 0.0167).

**FIGURE 8:**
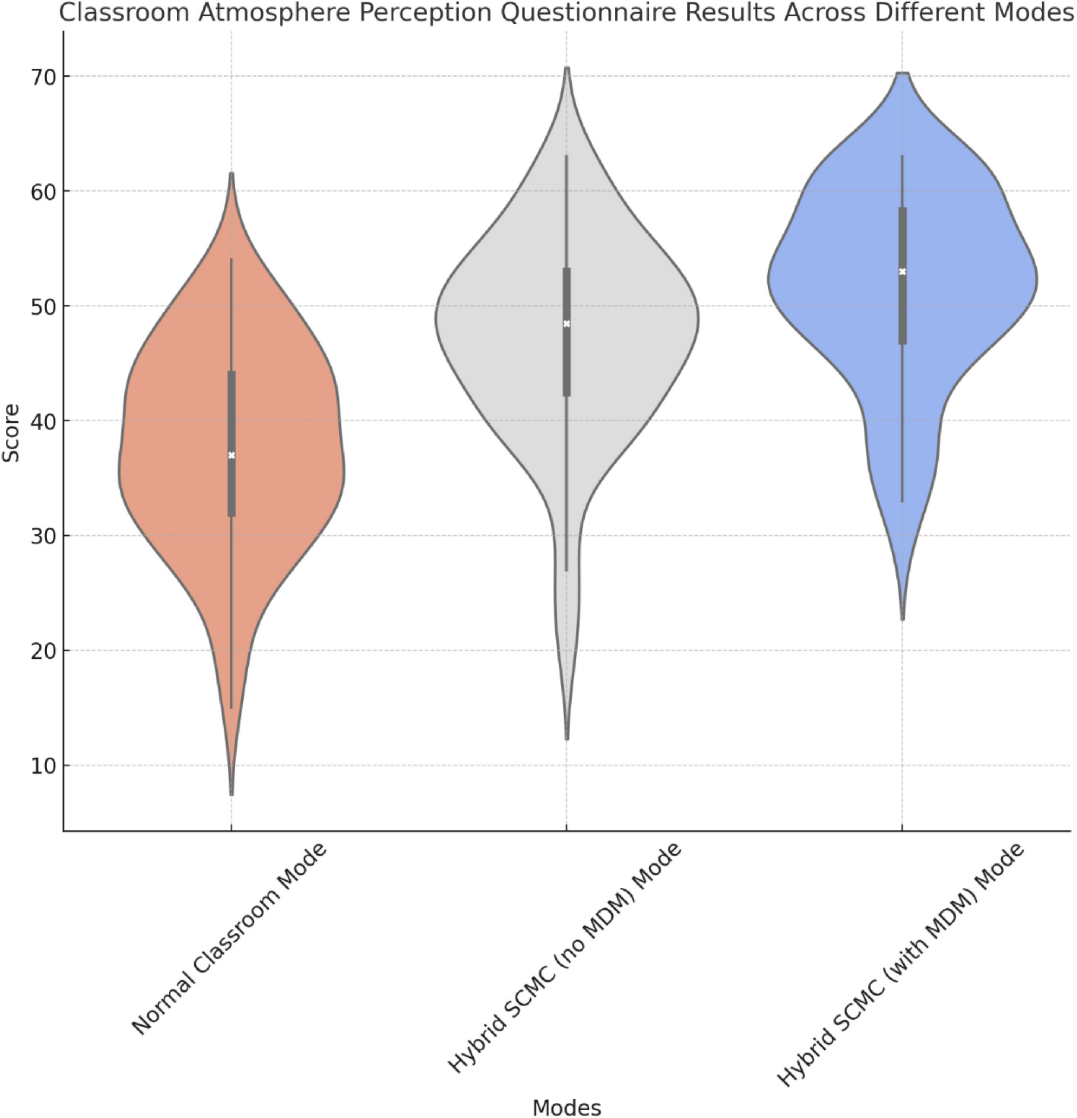
Graphical representation of the results regarding the classroom atmosphere.

These results indicate that the hybrid SCMC modes, especially the mode featuring MDM technology, significantly improved the classroom environment and atmosphere compared to the normal mode. Students rated the classroom environment and atmosphere significantly better in the hybrid SCMC modes than in the normal mode, suggesting that both hybrid SCMC approaches effectively enhanced classroom dynamics and engagement (Figure 8 and Table 3). The additional improvement observed with the inclusion of MDM technology highlights its potential value in further fostering a positive classroom atmosphere. Further research is needed to determine whether these effects are consistent across different educational contexts and with varying implementations of MDM technology.

**TABLE 3:** Normality test, Friedman test, and Pairwise Wilcoxon Signed-Rank comparisons of classroom-atmosphere scores (*N* = 60).

Normality Test (Shapiro–Wilk).			
Mode	Shapiro–Wilk Statistic	*p*-value	
Normal	0.9857	0.7075	
Hybrid SCMC (no MDM)	0.9591	0.0423	
Hybrid SCMC (with MDM)	0.9507	0.0168	
**Friedman test**			
**Test statistic**	**df**	**Kendall’s W**	***p*-value**
χ^2^(Friedman) = 52.361	2	0.507	<0.0001
**Pairwise Wilcoxon Signed-Rank Tests (Bonferroni-adjusted α = 0.0167)**			
**Comparison (A vs. B)**	***z***	**p (two-tailed)**	**Mean difference (A–B)**
Normal vs. Hybrid SCMC (no MDM)	–5.606	0.0000	–9.5000
Normal vs. Hybrid SCMC (with MDM)	–6.009	0.0000	–13.9167
Hybrid SCMC (no MDM) vs. Hybrid SCMC (with MDM)	–3.781	0.0002	–4.4167

#### 4.3.2 Qualitative analysis of open-ended responses to the classroom atmosphere perception questionnaire

A total of 51 valid responses were collected from the open-ended question associated with the Classroom Atmosphere Perception Questionnaire. These textual responses were analyzed using a qualitative content analysis approach.

Through iterative coding, seven recurring codes were identified and organized into three higher-order themes, reflecting dominant patterns as well as contrasting individual experiences related to classroom atmosphere across instructional modes.

##### 4.3.2.1 Theme 1: a more relaxed and participatory classroom atmosphere in hybrid SCMC modes

This dominant theme reflects students’ shared perceptions that both hybrid SCMC modes fostered a more relaxed, engaging, and supportive classroom atmosphere than the traditional classroom.

- A more relaxed classroom atmosphere in both hybrid SCMC modes (18 responses):
  Students frequently reported feeling more comfortable, enthusiastic, and willing to engage in learning activities in hybrid SCMC environments.
- Improved interaction in both hybrid SCMC modes (9 responses):
  The opportunity to respond through typed input reduced participation pressure and encouraged more students to contribute, resulting in a more lively and inclusive classroom atmosphere.

Together, these responses suggest that reduced performance pressure and mediated participation played a central role in shaping positive classroom atmosphere in hybrid SCMC modes.

##### 4.3.2.2 Theme 2: enhanced focus and self-regulation in the MDM-supported mode

This theme reflects a substantial proportion of student responses emphasizing the role of enhanced focus and self-discipline in shaping classroom atmosphere in the MDM-supported hybrid SCMC mode.

- Enhanced focus and self-discipline in the hybrid SCMC (with MDM) mode (21 responses):
  Students reported that clear usage rules and reduced off-task behavior helped maintain classroom order and attentional focus, which they perceived as beneficial for sustaining a productive and stable learning atmosphere.

This theme represents a salient secondary pattern in the qualitative data, indicating that for many students, structured technological regulation contributed positively to the perceived classroom atmosphere.

##### 4.3.2.3 Theme 3: divergent and contrasting perceptions of classroom atmosphere in new technology-supported modes

In contrast to the dominant patterns, a smaller set of responses expressed divergent or critical views, particularly toward the hybrid SCMC mode incorporating MDM.

- Effectiveness of the normal classroom mode (2 responses):
  Some students perceived the traditional classroom as more effective in fostering face-to-face interaction and interpersonal connection, noting that online-mediated participation in hybrid SCMC modes reduced opportunities for direct verbal communication.
- The MDM mode was excessively strict (1 response):
  A small number of students reported that the restrictive interface of the MDM-supported mode induced pressure or distraction, as they became preoccupied with navigating or bypassing constraints rather than focusing on instructional content.
- No significant differences across modes (5 responses):
  Several students reported perceiving little difference in classroom atmosphere among the three instructional modes.

While these views highlight potential limitations of technology-supported interaction, they can be interpreted within a broader pedagogical context. Although hybrid SCMC modes may reduce the frequency of face-to-face responses, they also appear to lower psychological barriers for students who typically avoid verbal participation. For these learners, responding through typed input represents a less demanding entry point into classroom interaction, which may help build confidence and serve as a stepping stone toward more challenging forms of communication. As face-to-face participation remains accessible within hybrid SCMC environments, reduced reliance on verbal responses does not necessarily preclude direct interaction but may instead facilitate gradual progression toward it.

From an instructional design perspective, these findings suggest that hybrid SCMC modes could be further optimized by incorporating explicit incentives or scaffolds (e.g., participation credit or bonus points) to encourage students to transition from online-mediated responses to face-to-face communication, thereby balancing inclusivity with interpersonal engagement.

#### 4.3.3 Interpretive considerations

The presence of divergent perceptions toward the hybrid SCMC (with MDM) mode suggests two complementary interpretations. First, these differences may reflect individual variability in adaptation to newly introduced instructional technologies, particularly given the relatively short 2-week implementation period. This interpretation aligns with the quantitative findings, in which response distributions for the latter two instructional modes deviated from normality, indicating heterogeneous learner experiences.

Second, the contrasting responses point to a tension between attentional control and perceived autonomy. While MDM-supported structures may enhance order and focus for many students, the same restrictions may be experienced as overly rigid by others, potentially constraining spontaneous interaction and perceived freedom. Similar patterns were observed in the open-ended responses to the Anxiety Perception Questionnaire (section 4.2.2), suggesting that such tensions extend across both affective and classroom atmosphere dimensions.

#### 4.3.4 Quantitative alignment

The qualitative themes align with the quantitative classroom-atmosphere results reported in section 4.3.1. Both hybrid SCMC modes were rated significantly higher than the normal classroom, and the MDM-supported condition showed an additional statistically significant improvement. The substantial number of responses describing a more relaxed atmosphere (18 cases) and enhanced focus in the MDM-supported mode (21 cases) corresponds to this pattern. Minority views emphasizing traditional face-to-face interaction or perceived strictness of MDM reflect individual variability rather than a reversal of the overall quantitative trend.

#### 4.3.5 Summary

Overall, the qualitative analysis indicates that students generally perceived hybrid SCMC modes as fostering a more relaxed, interactive, and supportive classroom atmosphere than the traditional classroom. The MDM-supported mode, in particular, was frequently associated with enhanced focus and self-regulation, contributing to a more orderly learning environment. At the same time, students’ responses reveal meaningful individual differences, especially regarding preferences for face-to-face interaction and perceptions of technological restriction. These findings underscore the importance of balancing structure, autonomy, and interactional diversity when designing technology-supported instructional modes aimed at optimizing classroom atmosphere.

### 4.4 Analysis of responses to the *smartphone internet-related distraction perception questionnaire*

The Smartphone Internet-Related Distraction Perception Questionnaire was administered after each instructional condition to examine learners’ perceived distraction across modes (see Supplementary Appendix 4). To assess measurement stability, reliability and factor structure were analyzed separately for each condition based on responses from 60 participants. The questionnaire demonstrated acceptable to high internal consistency, with Cronbach’s alpha values of 0.872 (normal), 0.909 (no MDM), and 0.818 (with MDM). Exploratory factor analyses further supported structural coherence, with KMO values ranging from 0.762 to 0.902 and significant Bartlett’s tests in all cases. Overall, the results indicate stable measurement performance across instructional conditions, supporting valid comparisons of perceived distraction.

#### 4.4.1 Quantitative analysis of smartphone internet-related distraction results

Quantitative responses were analyzed using repeated-measures ANOVA to examine differences in perceived smartphone internet-related distraction across instructional modes. The results revealed a significant main effect of instructional mode, with the hybrid SCMC mode supported by MDM demonstrating the lowest levels of perceived distraction among the three conditions.

##### 4.4.1.1 Normality test

All three instructional modes met the normality assumption (*p* = 0.694, 0.215, and.057), allowing the use of repeated measures ANOVA.

##### 4.4.1.2 RM ANOVA

The omnibus test showed a significant main effect of instructional mode on distraction scores, *F*(2, 118) = 152.17, *p* < 0.001, with a large effect size (partial η^2^ = 0.72).

##### 4.4.1.3 Post-hoc comparisons

Pairwise comparisons were conducted using a Bonferroni-corrected significance threshold (α≈ 0.0167).

- Normal vs. Hybrid SCMC (no MDM): significant difference (*p* = 0.005 < 0.0167).
- Normal vs. Hybrid SCMC (with MDM): significant difference (*p* = 0.0000 < 0.0167).
- Hybrid SCMC (no MDM) vs. Hybrid SCMC (with MDM): significant difference (*p* = 0.0000 < 0.0167).

**FIGURE 9:**
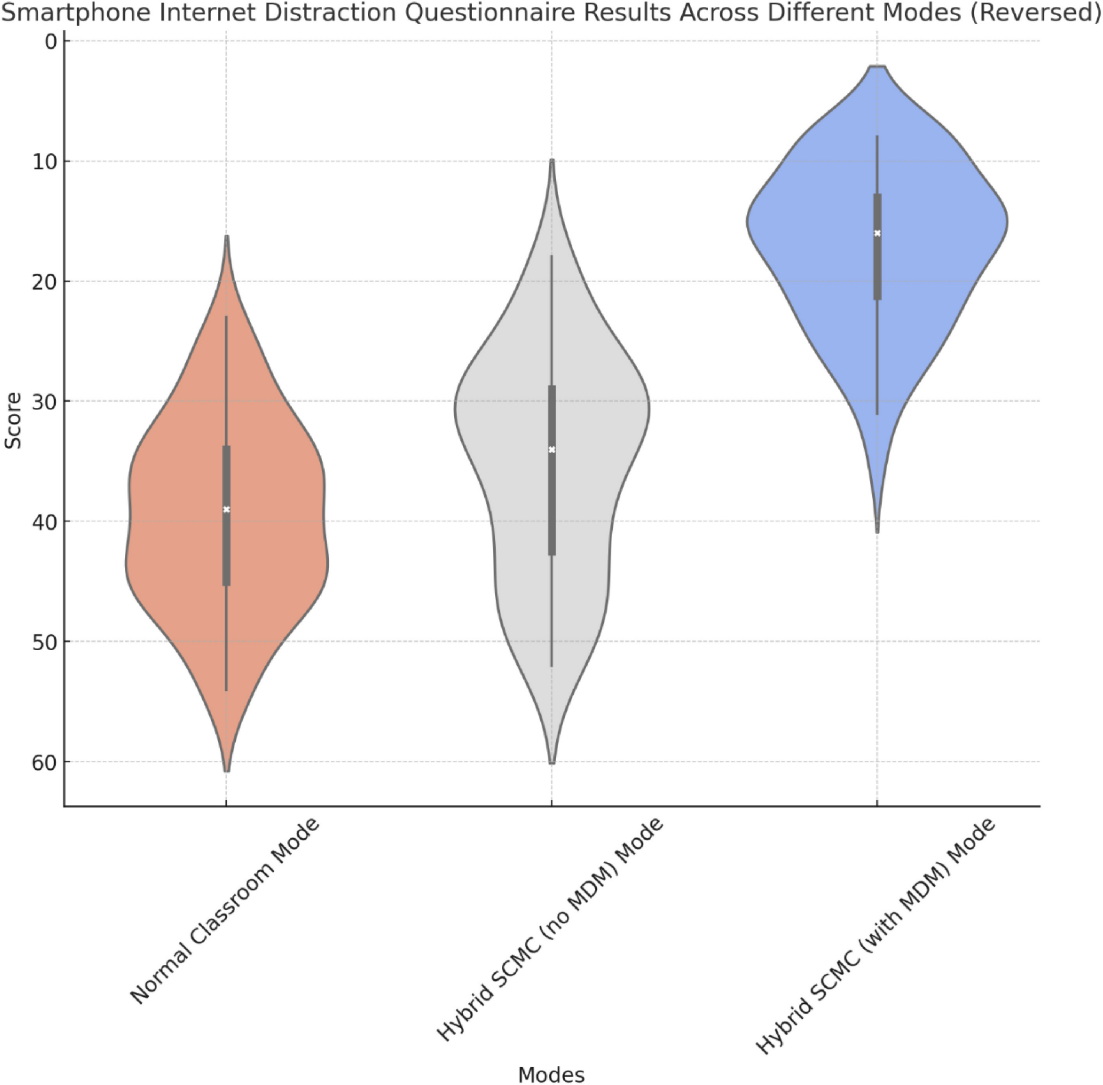
Graphical representation of the results regarding smartphone internet-related distraction.

Compared to the other two modes, the hybrid SCMC mode with MDM significantly reduced reported distraction. However, distraction levels reported in the hybrid SCMC mode without MDM differed from those reported in a previous study, which warrants further examination (Figure 9 and Table 4).

**TABLE 4:** Normality test, repeated measures ANOVA, and *post-hoc* comparisons of distraction scores (*N* = 60).

Normality test (Shapiro–Wilk)							
Mode				Shapiro–Wilk W	*p*-value		
Normal				0.9855	0.694		
Hybrid SCMC (no MDM)				0.9735	0.215		
Hybrid SCMC (with MDM)				0.9617	0.057		
**Repeated measures ANOVA**							
**Source**	**df**	***F***	***p***	**partial η^2^**			
Mode	2,118	152.17	<0.001	0.72			
***Post-hoc* comparisons of marginal means (Bonferroni-adjusted)**							
**Comparison (A vs. B)**					**Mean difference (A–B)**	**95% CI**	***p***
Normal vs. Hybrid SCMC (no MDM)					4.383	1.08–7.69	0.005
Normal vs. Hybrid SCMC (with MDM)					22.400	19.09–25.71	0.0000
Hybrid SCMC (no MDM) vs. Hybrid SCMC (with MDM)					18.017	14.71–21.32	0.0000

Huynh–Feldt correction applied (ε = 0.93).

In a previous study, distraction was assessed using a single, intuitive voting-based question, providing a coarse estimate of learners’ perceived distraction. In contrast, the present study employed a more detailed eight-item, seven-point Likert-scale questionnaire to capture multiple aspects of smartphone-related distraction. This more fine-grained measurement approach may have produced response patterns that differ from those reported in earlier work, reflecting differences in measurement resolution.

Although the questionnaire was designed to assess perceived distraction, some items did not explicitly distinguish between smartphone use for instructional purposes and non-educational use. In hybrid SCMC contexts, where mobile devices were actively integrated into learning activities, learners’ judgments of distraction may therefore have been influenced by their overall classroom experience. This represents a limitation in construct operationalization and suggests the need for further refinement of distraction measures to more precisely capture off-task mobile use in technology-enhanced classrooms.

#### 4.4.2 Qualitative analysis of open-ended responses on smartphone internet-related distraction

A total of 51 valid responses were collected from the open-ended section of the Smartphone Internet-Related Distraction Perception Questionnaire. These responses were analyzed using a qualitative content analysis approach.

Seven recurring codes were identified and further organized into three higher-order themes. Representative excerpts are provided to illustrate each category, and the number of similar responses is indicated in parentheses.

##### 4.4.2.1 Theme 1: perceived reduction of noneducational distraction through MDM control

This theme reflects students’ perceptions that MDM effectively restricts access to non-learning-related content and external notifications, thereby reducing distraction during class.

- Blocking noneducational internet use (7 responses):
*“When MDM is used, I don’t get any notifications from social media or games, which really helps me stay focused on learning during class.”*
- Tendency toward learning-related phone use (20 responses):
  Students reported that the hybrid SCMC (with MDM) mode encouraged them to use their smartphones primarily for class-related purposes, such as searching for information or completing assigned tasks.
- Comparative limitations of non-MDM modes (11 responses):
  Students frequently contrasted the MDM mode with normal and hybrid SCMC (no MDM) modes, noting that unrestricted access in these modes made it easier to switch to unrelated applications and become distracted.

##### 4.4.2.2 Theme 2: conditional effectiveness and the role of individual self-regulation

This theme captures students’ recognition that distraction is not determined solely by technological control, but is also influenced by individual factors and contextual conditions.

- Comprehensive and conditional evaluations of distraction (14 responses):
  In the normal mode, students reported occasional smartphone-related distraction. In the hybrid SCMC mode, phones were mainly used for teacher interaction, while in the MDM-supported mode, distracting notifications were largely blocked. Although motivation increased in the hybrid SCMC modes, smartphone use could still cause distraction. Overall, distraction in the MDM-supported mode was perceived as more controlled, though not entirely eliminated.
- Role of self-control (1 response):
*“MDM helps control phone use, but it doesn’t completely prevent distractions. Self-control is still very important.”*
- Perceived lack of difference across modes (1 response):
  A small number of students reported noticing little difference in distraction across instructional modes.

##### 4.4.2.3 Theme 3: residual distraction from platform-level notifications

A small number of responses pointed to residual sources of distraction originating from the SCMC platform itself, even under MDM control. Specifically, one student reported receiving internal push notifications and embedded video content generated by the platform software (e.g., *Superstar Learn*) during the hybrid SCMC (with MDM) mode.

Although these notifications were generally related to course content (such as reminders, updates, or instructional media), students noted that their appearance during class could momentarily interrupt attention. Device inspection of the collected COPE smartphones further indicated that, because the MDM system was not specifically designed for classroom teaching and the SCMC platform had not been fully optimized for MDM-supported instruction, certain built-in functions (e.g., internal chat groups or in-app advertisements) remained accessible. These features represent potential gaps in distraction management at the platform level.

##### 4.4.2.4 Quantitative Alignment

The qualitative findings are consistent with the quantitative results (section 4.4.1), which showed the strongest effect of instructional mode on distraction. Students’ reports of blocked noneducational access and task-oriented phone use in the MDM-supported mode correspond directly to the statistically significant reduction in distraction scores observed in that condition.

##### 4.4.2.5 Summary

Overall, the qualitative analysis of open-ended responses indicates that students generally perceived the hybrid SCMC mode with MDM as effective in reducing smartphone-related distraction by blocking non-learning-related internet access and encouraging task-oriented phone use. However, the findings also reveal conditional and residual forms of distraction, shaped by individual self-regulation and platform-level notification mechanisms. While MDM substantially improves attentional control in the classroom, its effectiveness is partly constrained by software design features, suggesting that closer integration between educational platforms and classroom-oriented MDM systems is necessary to further optimize distraction management.

### 4.5 Responses to the overall learning experience perception questionnaire

The Overall Learning Experience Perception Questionnaire was used to examine students’ perceived learning experience across the three instructional conditions: traditional classroom, hybrid SCMC without MDM, and hybrid SCMC with MDM (see Supplementary Appendix 5). To assess measurement stability, reliability and factor structure were analyzed separately for each condition based on responses from 60 participants. The questionnaire demonstrated high internal consistency across modes, with Cronbach’s alpha values of 0.965 (normal), 0.928 (no MDM), and 0.965 (with MDM). Exploratory factor analyses further supported structural coherence, with KMO values ranging from 0.888 to 0.926 and significant Bartlett’s tests in all cases (χ^2^ = 675.045–779.380). These results indicate stable reliability and structural consistency across instructional conditions, supporting valid cross-mode comparisons of overall learning experience.

#### 4.5.1 Normality test

Only the normal mode met the normality assumption (*p* = 0.5995), whereas both hybrid SCMC modes violated normality (*p* = 0.0046 and 0.0028), requiring the use of non-parametric tests.

#### 4.5.2 Friedman test

The Friedman test revealed a significant overall difference in overall learning-experience scores across the three instructional modes, χ^2^(2) = 76.816, *p* < 0.0001, with a moderate-to-large effect size (Kendall’s *W* = 0.426).

#### 4.5.3 *Post-hoc* comparisons

Pairwise comparisons were conducted using a Bonferroni-corrected significance threshold (α≈ 0.0167).

- Normal vs. Hybrid SCMC (no MDM): significant (*p* < 0.0167).
- Normal vs. Hybrid SCMC (with MDM): significant (*p* < 0.0167).
- Hybrid SCMC (no MDM) vs. Hybrid SCMC (with MDM): significant (*p* < 0.0167).

**FIGURE 10:**
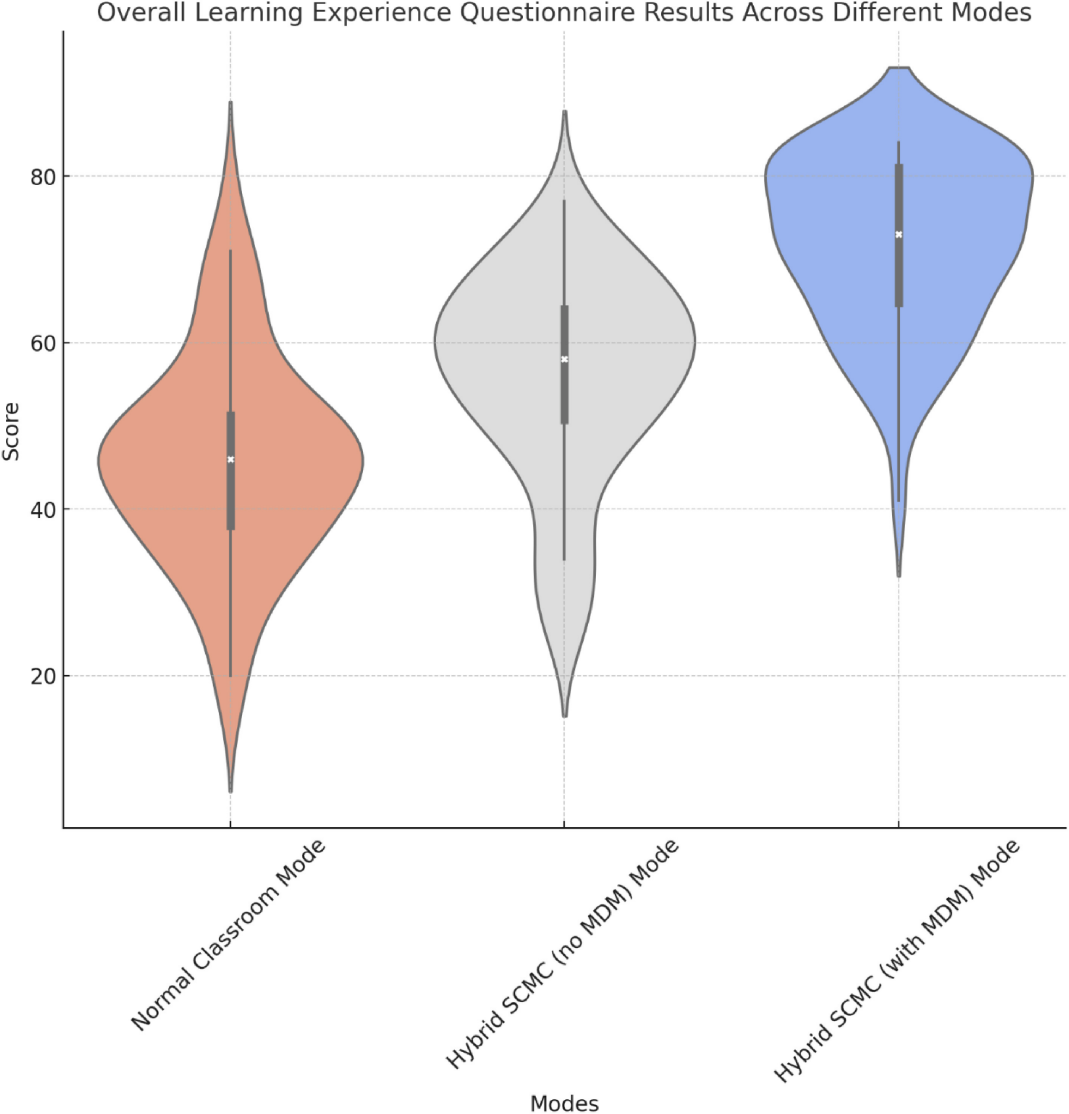
Graphical representation of the results regarding the overall learning experience.

The results indicate that hybrid SCMC modes significantly improved the overall learning experience. The hybrid mode with MDM further enhanced students’ learning experience by establishing a more structured and focused environment that was conducive to improvements in interaction quality and perceived learning experience (Figure 10 and Table 5).

**TABLE 5:** Normality test, Friedman test, and Pairwise Wilcoxon Signed-Rank comparisons of overall learning-experience scores (*N* = 60).

Normality test (Shapiro–Wilk)						
Mode		Shapiro–Wilk Statistic		*p*-value		
Normal		0.9836		0.5995		
Hybrid SCMC (no MDM)		0.9383		0.0046		
Hybrid SCMC (with MDM)		0.9333		0.0028		
**Friedman test**						
**Test statistic**	**df**	**Kendall’s W**	***p*-value**			
χ^2^(Friedman) = 76.816	2	0.426	<0.0001			
**Pairwise Wilcoxon Signed-Rank Tests (Bonferroni-adjusted α = 0.0167)**						
**Comparison (A vs. B)**				***z***	***p* (two-tailed)**	**Mean difference (A–B)**
Normal vs. Hybrid SCMC (no MDM)				–4.806	0.0000	–10.3000
Normal vs. Hybrid SCMC (with MDM)				–6.524	0.0000	–26.1500
Hybrid SCMC (no MDM) vs. Hybrid SCMC (with MDM)				–6.312	0.0000	–15.8500

Only the normal mode met the normality assumption; both hybrid SCMC modes showed significant deviations from normality. The Friedman test indicated a significant overall difference among the three modes, with Kendall’s *W* ≈ 0.43 indicating a moderate-to-large effect.

With the exception of the FLCAS, all the scales used in this study were self-designed. In the actual testing process, the results regarding the FLCAS consistently conformed to a normal distribution. However, with respect to the self-designed perception questionnaires, in three out of 4 of them (i.e., anxiety, atmosphere, and overall learning experience; the exception was distraction), only the results concerning the normal mode exhibited a normal distribution, whereas those concerning the two technology-supported modes did not. This discrepancy could be attributed to the fact that students were familiar with the normal mode beyond the experimental context, whereas they experienced the technology-supported modes for only 2 weeks, thus leading to varying adaptation rates among students as well as internal inconsistencies despite the significant findings of this research. Additionally, the FLCAS is more established; for example, it includes 20 questions, as compared to the approximately 10 questions included in the perception questionnaires. Future researchers should consider extending the duration of exposure to the technology-supported modes and refining the questionnaires to increase their reliability.

## 5 Discussion—what MDM adds on top of hybrid SCMC

### 5.1 What this study adds about MDM

#### 5.1.1 Anxiety

Both hybrid conditions alleviated foreign-language anxiety relative to the traditional classroom, aligning with prior findings that mediated turn-taking and additional planning time reduce face-threatening immediacy. The addition of MDM did not further reduce anxiety, suggesting that anxiety relief is primarily driven by the communicative affordances of SCMC rather than by device governance.

This pattern may be understood in terms of functional differentiation. SCMC directly alters the interactional structure of classroom communication by reducing immediacy and public exposure, thereby addressing core sources of foreign-language anxiety. In contrast, MDM primarily operates at the level of attentional regulation by limiting off-task digital access and suppressing competing media stimuli. While such regulation enhances focus and classroom atmosphere, it does not directly modify the social-evaluative dynamics that typically trigger language anxiety.

At the same time, it remains possible that MDM contributes indirectly to emotional experience through attentional stabilization. By reducing distraction, MDM may help preserve the cognitive and participatory benefits created by SCMC, even if this effect does not manifest as an additional statistically significant reduction in anxiety scores. This interpretation is supported by the qualitative findings in section 4.2.2 (Theme 2), where several students reported feeling more settled and focused under the MDM-supported condition. These responses suggest that attentional regulation may enhance subjective learning stability, although it does not directly alter the social-evaluative dynamics that primarily drive foreign-language anxiety. Future research employing mediation or longitudinal designs could further examine whether attentional regulation exerts downstream effects on affective outcomes over extended periods.

Beyond statistical differences, the manifestation of anxiety appears qualitatively distinct across instructional contexts. In the traditional classroom, anxiety was primarily associated with immediate face-to-face exposure, fear of negative evaluation, and pronunciation concerns. In contrast, the hybrid SCMC modes altered the communicative format by enabling text-based participation, which reduced immediacy, allowed additional planning time, and minimized public scrutiny. Although not fully anonymous, the mediated nature of text interaction appears to attenuate perceived social pressure, thereby increasing students’ willingness to express themselves.

Within this dynamic, MDM does not fundamentally modify the communicative structure that shapes social-evaluative anxiety. Rather, it operates at the level of attentional governance. While improved focus may contribute to a calmer learning atmosphere, the present findings suggest that anxiety reduction is primarily attributable to the mode of communication rather than to device regulation. In this sense, MDM does not moderate the communicative affordance of SCMC but instead supports the stability of the learning environment in which that affordance operates.

#### 5.1.2 Classroom atmosphere

Both hybrid modes improved perceived classroom atmosphere compared with the traditional mode, with the MDM-assisted condition producing an additional, statistically reliable gain. Students attributed this improvement to more disciplined device use, which reduced background digital noise and enabled quieter learners to participate more consistently. Psychologically, these findings suggest that MDM contributes to classroom atmosphere by supporting shared attentional norms rather than by altering emotional states directly. In this sense, SCMC opens the participatory space, while MDM helps maintain its psychological coherence.

#### 5.1.3 Engagement tension: focus and autonomy

The qualitative findings also reveal a psychological tension underlying MDM implementation. On the one hand, many students associated MDM-supported regulation with enhanced focus, self-discipline, and a more orderly classroom atmosphere. From this perspective, structured device governance may scaffold self-regulation by reducing environmental distractions and clarifying attentional norms. On the other hand, a minority of students experienced the same restrictions as limiting or overly strict, suggesting that externally imposed control may reduce perceived autonomy for certain learners.

This dual pattern indicates that MDM’s impact on engagement is not unidirectional. While attentional regulation can support sustained participation and classroom coherence, excessive or inflexible restriction may generate resistance or psychological reactance in some students. Future research could explore adaptive MDM strategies that balance structure with autonomy—for example, incorporating controlled flexibility (e.g., limited search windows or student-managed task modes) to maintain focus while preserving a sense of agency.

#### 5.1.4 Distraction control

Distraction control represents the domain in which MDM exerts its strongest psychological effect. Restricting non-instructional applications and notifications reduced off-task switching and temptation, with students reporting fewer interruptions and less mind-wandering. These results indicate that MDM primarily operates by constraining competing media affordances, thereby supporting sustained attentional engagement during learning activities.

#### 5.1.5 Overall learning experience

The hybrid SCMC with MDM condition received the highest overall learning-experience ratings. A plausible psychological pathway is indirect: SCMC enhances participation and psychological safety, while MDM suppresses digital distraction; together, these conditions free attentional resources for learning. This interaction helps explain why MDM contributes more strongly to perceived atmosphere and engagement than to anxiety reduction.

#### 5.1.6 Mechanism in brief

Taken together, the findings support a two-layer psychological account: (1) SCMC reduces social-evaluative threat and facilitates planned participation; (2) MDM regulates attention by constraining the notification and application landscape. This division of psychological labor explains why MDM primarily influences classroom atmosphere, distraction control, and overall experience rather than foreign-language anxiety.

#### 5.1.7 Psychological implications for practice

From a psychological standpoint, the following design principles translate the observed mechanisms into classroom practice. Importantly, MDM should be implemented as a supportive attentional scaffold rather than as a rigid control system. The goal is to enhance focus and engagement while maintaining a positive and autonomy-supportive learning climate:

- Whitelist for the task. Allow only tools necessary for the learning activity (e.g., SCMC platform, LMS, dictionary), directly targeting the attentional leakage pathway identified in this study.
- Suppress in-platform push. Select platforms or settings that minimize in-app notifications during class, addressing sources of distraction beyond generic system-level control.
- Plan for BYOD–COPE hybrids. Where operating-system restrictions limit control on personal devices, maintaining a small pool of institution-managed devices can ensure consistent attentional regulation without intrusive intervention.

In addition to technical configuration, implementation strategy is crucial. Educators may consider activating MDM restrictions primarily during focused task phases while allowing limited flexibility during discussion or exploratory activities. Transparent communication about the pedagogical purpose of MDM can further reduce perceptions of surveillance or excessive restriction. Such calibrated use of MDM may help balance attentional structure with students’ sense of autonomy, thereby sustaining both engagement and classroom trust.

### 5.2 Research significance

The present study addresses a fundamental challenge in contemporary technology-enhanced education. While a wide range of digital technologies—including computers, mobile devices, networked platforms, and emerging AI-supported tools—offer compelling pedagogical opportunities, their classroom integration is often accompanied by substantial psychological costs. Among these, digital distraction arising from social media, online games, and short-form video platforms represents one of the most persistent negative influences on learners’ attention and engagement. Without effective mechanisms to manage such distraction, the educational potential of new technologies may remain only partially realized, resulting in mixed or suboptimal instructional outcomes.

Using hybrid synchronous computer-mediated communication (SCMC) as a representative form of computer-assisted instruction, the present study demonstrates that while mediated interaction itself affords affective benefits—such as reduced anxiety and a more supportive interactional climate—these benefits may be compromised in mobile-supported classrooms by unmanaged digital distraction. Rather than directly enhancing affective outcomes, Mobile Device Management (MDM) functions as an attentional regulation mechanism that constrains sources of distraction and prevents the erosion of SCMC’s existing emotional and interactional advantages. By integrating communicative affordances with attention-sensitive governance, this study shows how learners are better able to sustain engagement and realize the instructional potential of technology-mediated interaction without the disruptive influence of off-task mobile use.

From a theoretical perspective, this study contributes to the literature by empirically disentangling the complementary roles of interactional design and attentional governance in technology-mediated learning. Previous SCMC research has predominantly focused on communicative processes and affective outcomes under controlled or predominantly online instructional conditions (e.g., Tudini, 2007; York et al., 2021; Kobayashi, 2025), which limits ecological validity. Although Liu (2023) extended hybrid SCMC research into authentic classroom practice, challenges related to smartphone use and internet-based distraction remained insufficiently addressed, and no regulatory mechanism such as MDM was incorporated. In contrast, the present study situates hybrid SCMC within real classroom settings and explicitly integrates MDM to address attentional interference, thereby extending existing SCMC research from interactional facilitation to attention-sensitive instructional design.

In addition, this study advances research on Mobile Device Management by moving beyond predominantly conceptual discussions or non-MDM-focused empirical work (e.g., Emery, 2012; Samochadin et al., 2015; Attah et al., 2025; Martin et al., 2025), as well as descriptive, school-level case reports based on professional reflections and institutional outcomes (e.g., Smith, 2016). Rather than treating MDM solely as a technical or administrative tool, the present research applies psychological research methods to provide systematic learner-level evidence on how regulated device use shapes attentional focus, emotional experience, and perceived learning quality within technology-rich instructional environments. This perspective addresses a critical gap in the existing literature, where direct psychological measurement of MDM effects has been largely absent.

From an applied perspective, the significance of the proposed hybrid SCMC–MDM framework extends beyond foreign language education. Many instructional domains face comparable challenges related to classroom interaction, learner anxiety, and sustained engagement in technology-rich learning environments. By combining mediated interaction with structured attentional governance, the hybrid SCMC–MDM approach offers a transferable model for supporting focused participation across disciplines. More broadly, the findings provide early empirical guidance for the responsible integration of computer-, mobile-, and AI-assisted instructional technologies, suggesting that the pedagogical potential of emerging tools can be more fully realized when their psychological costs are systematically addressed rather than left unmanaged.

### 5.3 Limitations

This study has several limitations. First, although a 3 × 3 counterbalanced self-contrast design was employed to reduce potential sequence effects, subtle carry-over influences between instructional modes cannot be fully excluded. Experiencing one mode may have influenced how students perceived subsequent modes. Nevertheless, the counterbalanced arrangement has been used to mitigate systematic order bias.

Second, to enable participants to make explicit comparisons among the three instructional modes, the perception questionnaires were administered once—after all modes had been experienced. This design minimized practice effects and survey fatigue and offered a comprehensive comparative perspective; however, it may have reduced the immediacy of certain impressions. Future studies could incorporate post-mode assessments to capture more time-proximal perceptions.

In addition, although each instructional mode was implemented for 2 weeks, this duration may not have been sufficient for all learners to fully adapt to the two technology-enhanced modes. Individual differences in familiarity with and acceptance of new instructional technologies may have contributed to response variability, particularly in the hybrid SCMC conditions. The study did not independently profile participants’ prior exposure to mobile learning environments or stable individual differences in foreign language anxiety. Such factors may have shaped how students experienced the instructional modes. Given the sample size (*N* = 60), these individual differences may have contributed to variation in perception measures. Importantly, this variation reflects learner heterogeneity rather than measurement instability.

Furthermore, the distraction questionnaire, while demonstrating satisfactory reliability and structural consistency, may not have fully distinguished between non-educational smartphone use and task-related mobile use in hybrid SCMC contexts. This construct-operationalization limitation may have influenced how learners evaluated perceived distraction. Future research could address these issues by employing larger samples, extending exposure duration, incorporating baseline profiling measures, and further refining questionnaire items.

Finally, the MDM software employed in this study was not purpose-built for educational contexts. Certain distractions, such as push notifications or in-app advertisements, could still occur, potentially limiting the maximum achievable level of distraction control. Future research could benefit from collaboration with vendors to develop education-specific “class mode” features.

### 5.4 Directions for future research

Future research should further investigate how media regulation mechanisms interact with attention, emotion, and engagement over extended periods and across diverse learning contexts. As AI- and internet-supported instruction becomes increasingly common, understanding how technological constraints shape learners’ psychological experiences will be essential for designing environments that support sustained attention and meaningful interaction.

## 6 Conclusion

This study examined the incremental value of adding a mobile device management (MDM) layer to hybrid synchronous computer-mediated communication (SCMC) by focusing on the psychological processes through which technological conditions influence classroom experience. Rather than re-establishing whether hybrid SCMC is effective, the findings clarify how attentional regulation helps it function more reliably in real classroom settings. Pairing MDM with hybrid SCMC produced a more focused and psychologically coherent learning environment and higher overall learning experience, while the anxiety reduction associated with hybrid SCMC itself did not meaningfully increase with additional device controls.

From a practical perspective, these results point to a clear division of psychological labor. Hybrid SCMC primarily widens participation by reducing social-evaluative threat, whereas MDM contributes by constraining competing media affordances and protecting learners’ attention. Accordingly, hybrid SCMC can be adopted to address anxiety and participation, while targeted MDM configurations are most beneficial when learning activities are vulnerable to digital distraction, for example through task-specific whitelists and suppression of in-app push within the SCMC/LMS ecosystem. Where bring-your-own-device policies limit control, a small pool of institution-managed devices may provide more consistent attentional support.

These conclusions should be interpreted in light of the study’s scope, including its single-site design, short exposure periods, and use of a general-purpose MDM solution. As outlined in sections 5.3 and 5.4, future research should extend exposure duration, examine a broader range of disciplines and educational levels, and combine subjective and objective indicators to further test the durability of attentional regulation and its translation into learning performance.

In sum, this study reframes the contribution of classroom technology from a question of instructional effectiveness to one of psychological mediation. By demonstrating how classroom governance via MDM supports attentional stability in mobile-mediated interaction, the findings highlight how psychological processes serve as the pathway through which hybrid SCMC’s interactive potential is converted into more robust, distraction-resistant educational practice.

## Data Availability

The original contributions presented in this study are included in this article/Supplementary material, further inquiries can be directed to the corresponding author.

## Footnotes

1
https://www.statstodo.com/
