# Correction: Psychological effects of hybrid SCMC with mobile device management: distraction, classroom atmosphere, and foreign language anxiety

**DOI:** 10.3389/fpsyg.2026.1857095

**Published:** 2026-04-28

**Authors:** Xuecheng Liu, Jiabei Zhu

**Affiliations:** 1 Institute of Global Humanities, Nanjing University, Nanjing, China; 2 School of General Education, Wanjiang University of Technology, Ma'anshan, China

**Keywords:** mobile device management (MDM), hybrid SCMC, foreign language anxiety, digital distraction, classroom atmosphere, mobile learning, technology-mediated learning, attention regulation

In the abstract, the Results section omitted that overall learning experience was significantly enhanced in the hybrid SCMC with MDM condition. The original Results section was: “Results: The results indicate that hybrid SCMC with MDM significantly improves classroom focus and reduces digital distraction compared with the other conditions, whereas the direct effect of MDM on foreign language anxiety is limited.”

In addition, the Discussion section did not reflect the study's central mechanism. The original Discussion section was: “Discussion: These findings suggest that MDM shapes learners' psychological experiences primarily by regulating attention rather than directly modulating emotional responses. By clarifying how mobile media constraints shape learners' psychological experiences, particularly through the integration of communicative affordances and attentional regulation, this study contributes to understanding media-mediated interaction in educational contexts and highlights how psychological mechanisms can inform the design of more effective hybrid learning environments.”

This has been corrected to read:

**“Results:** The results indicate that hybrid SCMC with MDM significantly improves classroom focus, reduces digital distraction, and enhances overall learning experience compared with the other conditions. However, the direct effect of MDM on foreign language anxiety is limited.

**Discussion:** These findings suggest that MDM shapes learners' psychological experiences primarily by regulating attention rather than directly modulating emotional responses. MDM helps regulate smartphone-related distraction, thereby creating more favorable attentional conditions for the effective implementation of hybrid SCMC. By clarifying how mobile media constraints shape learners' psychological experiences, particularly through the integration of communicative affordances and attentional regulation, this study contributes to understanding media-mediated interaction in educational contexts and highlights how psychological mechanisms can inform the design of more effective hybrid learning environments.”

The original version of this article has been updated.

