# Supplementary material for: Psychological effects of hybrid SCMC with mobile device management: distraction, classroom atmosphere, and foreign language anxiety
Source: Front Psychol. 2026 Mar 25;17:1775057. doi: 10.3389/fpsyg.2026.1775057 (PMC13057340; doi:10.3389/fpsyg.2026.1775057)
Supplement: Supplementary file 1 [file Supplementary_file_1.docx]

**Appendix 1**

**Adapted Items Based on the Foreign Language Classroom Anxiety Scale (FLCAS)**

The following items were selected and adapted from the Foreign Language Classroom Anxiety Scale (FLCAS) developed by Horwitz, Horwitz, and Cope (1986). The original instrument contains 33 items. The present study includes 20 items relevant to classroom interaction in English-language learning contexts.

Horwitz, E. K., Horwitz, M., & Cope, J. (1986). *Foreign language classroom anxiety*. Modern Language Journal, 70, 125–132. <https://doi.org/10.1111/j.1540-4781.1986.tb05256.x>

Responses were measured on a 5-point Likert scale:

1 = Strongly disagree

2 = Disagree

3 = Neither agree nor disagree

4 = Agree

5 = Strongly agree

Note: Items 2, 6, and 11 were reverse-coded prior to analysis.

**Items Used in the Present Study**

1. I feel unsure about my ability when speaking English in class.
2. I am not concerned about making mistakes when speaking in class.
3. I feel nervous when I am called on to speak in class.
4. I feel anxious if I do not understand what the teacher says in English.
5. I often believe that my classmates speak English better than I do.
6. I feel at ease when taking short quizzes in English class.
7. I feel panicked when asked to speak without preparation.
8. I sometimes become so nervous in class that I forget what I know.
9. Volunteering to speak in English class makes me feel embarrassed.
10. I feel uneasy when I do not understand the teacher’s corrections.
11. I feel confident when speaking English in class.
12. I feel worried when the teacher corrects my mistakes.
13. My heart races when I am about to be called on.
14. I believe other students are more proficient in English than I am.
15. I feel self-conscious when speaking English in front of others.
16. I feel more tense in English class than in my other classes.
17. I feel nervous and confused when speaking English in class.
18. I feel uncomfortable if I do not understand every word the teacher says.
19. I worry that other students may laugh at me when I speak English.
20. I feel nervous when asked questions I have not prepared for.

**Appendix 2**

**The Survey Used to Compare Anxiety Levels in Foreign Language Learning across the Three Teaching Modes**

1. When answering questions in a foreign language class, I worry about making mistakes.

1 (Strongly Disagree) 2 3 4 5 6 7 (Strongly Agree)

- Normal classroom mode
- Hybrid SCMC (no MDM) mode
- Hybrid SCMC (with MDM) mode

1. When the teacher initiates a discussion topic, I don't dare to speak up unless the teacher calls on me.

1 (Strongly Disagree) 2 3 4 5 6 7 (Strongly Agree)

- Normal classroom mode
- Hybrid SCMC (no MDM) mode
- Hybrid SCMC (with MDM) mode

1. I am afraid of embarrassing myself in class due to my lack of fluency. (The options are the same as in questions 1 and 2)
2. I feel nervous and uneasy when I speak in a foreign language class. (The options are the same as in questions 1 and 2)
3. I fear being laughed at by classmates due to my foreign language skills when I answer questions. (The options are the same as in questions 1 and 2)
4. I am afraid of being called on when the teacher asks a question. (The options are the same as in questions 1 and 2)
5. I find the interactive activities in the foreign language class to be stressful. (The options are the same as in questions 1 and 2)
6. I feel extremely nervous when I am asked to answer questions in a foreign language class. (The options are the same as in questions 1 and 2)
7. After reflecting on your feelings and ratings, please first summarize in one to two sentences the differences in the levels of anxiety that you experience when learning in foreign language classes among the three modes (if any). Then, in two to three sentences, discuss the potential reasons underlying these differences from your perspective.
   - Open-ended response

**Appendix 3**

**The Survey Used to Compare the Classroom Atmosphere across the Three Teaching Modes**

1. In the current teaching mode, I feel that the classroom atmosphere encourages student participation and communication.

1 (Strongly Disagree) 2 3 4 5 6 7 (Strongly Agree)

- Normal classroom mode
- Hybrid SCMC (no MDM) mode
- Hybrid SCMC (with MDM) mode

1. The current teaching approach has a positive effect on the establishment of a positive classroom atmosphere.

1 (Strongly Disagree) 2 3 4 5 6 7 (Strongly Agree)

- Normal classroom mode
- Hybrid SCMC (no MDM) mode
- Hybrid SCMC (with MDM) mode

1. I feel that the current classroom setup (e.g., in terms of seating arrangements or teaching media) is conducive to learning. (The options are the same as in questions 1 and 2)
2. In the current teaching mode, I can receive support and encouragement from classmates and teachers. (The options are the same as in questions 1 and 2)
3. I believe that this teaching mode helps establish a learning environment characterized by mutual respect and support. (The options are the same as in questions 1 and 2)
4. I feel that there is a good atmosphere of interaction and collaboration in the classroom. (The options are the same as in questions 1 and 2)
5. The classroom atmosphere has stimulated my interest in learning and enthusiasm for participation. (The options are the same as in questions 1 and 2)
6. In this teaching mode, I can sense good relationships between my classmates and teachers. (The options are the same as in questions 1 and 2)
7. Overall, I am satisfied with the classroom environment and atmosphere in the current teaching mode. (The options are the same as in questions 1 and 2)
8. After reflecting on your feelings and ratings, please first summarize in one to two sentences the differences in the classroom atmosphere among the three modes (if any). Then, in two to three sentences, discuss the potential reasons underlying these differences from your perspective.
   - Open-ended response

**Appendix 4**

**The Survey Used to Compare the Impact of Mobile Internet on Classroom Learning Distractions across the Three Teaching Modes**

1. In the classroom, I often feel distracted by the use of the mobile internet.

1 (Strongly Disagree) 2 3 4 5 6 7 (Strongly Agree)

- Normal classroom mode
- Hybrid SCMC (no MDM) mode
- Hybrid SCMC (with MDM) mode

1. In the classroom, I have engaged in many activities that are unrelated to learning via the mobile internet.

1 (Strongly Disagree) 2 3 4 5 6 7 (Strongly Agree)

- Normal classroom mode
- Hybrid SCMC (no MDM) mode
- Hybrid SCMC (with MDM) mode

1. My primary reason for using my phone in the classroom is related to learning.

7 (Strongly Agree) 6 5 4 3 2 1 (Strongly Disagree)

- Normal classroom mode
- Hybrid SCMC (no MDM) mode
- Hybrid SCMC (with MDM) mode

1. I find that using the mobile internet in the classroom makes it difficult for me to focus on the teaching content. (The options are the same as in questions 1 and 2)
2. I become distracted in the classroom due to social media or messaging apps on my phone. (The options are the same as in questions 1 and 2)
3. Using my phone in the classroom causes me to miss important explanations or discussions. (The options are the same as in questions 1 and 2)
4. In the classroom, I feel that I can control myself from becoming distracted by social media or games on my phone.

7 (Strongly Agree) 6 5 4 3 2 1 (Strongly Disagree)

- Normal classroom mode
- Hybrid SCMC (no MDM) mode
- Hybrid SCMC (with MDM) mode

1. I feel that using the mobile internet in the classroom in the current mode has a negative impact on my learning effectiveness. (The options are the same as in questions 1 and 2)
2. After reflecting on your feelings and ratings, please first summarize in one to two sentences the differences in the impact of mobile internet use on classroom distractions among the three modes (if any). Then, in two to three sentences, discuss the potential reasons underlying these differences from your perspective.
   - Open-ended response

**Appendix 5**

**The Survey Used to Compare the Overall Learning Experience across the Three Teaching Modes**

1. In this teaching mode, I can focus on learning for a longer period.

1 (Strongly Disagree) 2 3 4 5 6 7 (Strongly Agree)

- Normal classroom mode
- Hybrid SCMC (no MDM) mode
- Hybrid SCMC (with MDM) mode

1. I feel that the interactive activities included in this teaching mode help enhance my understanding of the learning content.

1 (Strongly Disagree) 2 3 4 5 6 7 (Strongly Agree)

- Normal classroom mode
- Hybrid SCMC (no MDM) mode
- Hybrid SCMC (with MDM) mode

1. My level of participation in the classroom is high in this teaching mode. (The options are the same as in questions 1 and 2)
2. This teaching mode promotes effective interaction between students and teachers. (The options are the same as in questions 1 and 2)
3. I feel that my time in the classroom is spent effectively in this teaching mode. (The options are the same as in questions 1 and 2)
4. I am able to remain focused on teaching activities throughout the entire class period in this teaching mode. (The options are the same as in questions 1 and 2)
5. This teaching mode makes it easier for me to express my thoughts and opinions. (The options are the same as in questions 1 and 2)
6. I feel more immersed and engaged in the classroom in this teaching mode than in other such modes. (The options are the same as in questions 1 and 2)
7. I would recommend applying this teaching mode to other courses. (The options are the same as in questions 1 and 2)
8. The technical support provided in this teaching mode helps me engage in learning and classroom interactions more effectively. (The options are the same as in questions 1 and 2)
9. I believe that this teaching mode has improved my learning efficiency. (The options are the same as in questions 1 and 2)
10. Overall, I am satisfied with my learning experience in this teaching mode. (The options are the same as in questions 1 and 2)
